# Distinct Microbiotas Are Associated with Different Production Lines in the Cutting Room of a Swine Slaughterhouse

**DOI:** 10.3390/microorganisms11010133

**Published:** 2023-01-04

**Authors:** Fanie Shedleur-Bourguignon, Tom Duchemin, William P. Thériault, Jessie Longpré, Alexandre Thibodeau, Mounia N. Hocine, Philippe Fravalo

**Affiliations:** 1 NSERC Industrial Research Chair in Meat Safety (CRSV), Faculté de Médecine Vétérinaire, Université de Montréal, Saint-Hyacinthe, QC J2S 2M2, Canada; 2 MESuRS Laboratory (Modelling, Epidemiology and Surveillance of Health Risks), Conservatoire National des Arts et Métiers (Cnam), 75003 Paris, France; 3 F. Ménard, Division d’Olymel s.e.c., Ange-Gardien, QC J0E 1E0, Canada; 4 CRIPA Swine and Poultry Infectious Diseases Research Center, Faculté de Médecine Vétérinaire, Université de Montréal, Saint-Hyacinthe, QC J2S 2M2, Canada; 5 Le Conservatoire National des Arts et Métiers (Cnam), 75003 Paris, France

**Keywords:** production lines, 16S rRNA sequencing, random forest, cutting room, swine slaughterhouse

## Abstract

The microorganisms found on fresh, raw meat cuts at a slaughterhouse can influence the meat’s safety and spoilage patterns along further stages of processing. However, little is known about the general microbial ecology of the production environment of slaughterhouses. We used 16s rRNA sequencing and diversity analysis to characterize the microbiota heterogeneity on conveyor belt surfaces in the cutting room of a swine slaughterhouse from different production lines (each associated with a particular piece/cut of meat). Variation of the microbiota over a period of time (six visits) was also evaluated. Significant differences of alpha and beta diversity were found between the different visits and between the different production lines. Bacterial genera indicative of each visit and production line were also identified. We then created random forest models that, based on the microbiota of each sample, allowed us to predict with 94% accuracy to which visit a sample belonged and to predict with 88% accuracy from which production line it was taken. Our results suggest a possible influence of meat cut on processing surface microbiotas, which could lead to better prevention, surveillance, and control of microbial contamination of meat during processing.

## 1. Introduction

Providing safe and organoleptically satisfactory food for the consumer is a major concern for the food industry [1]. In Canada, 11 million tons of food produced each year are preventable waste and four million—or one in eight people—become sick every year from eating contaminated food [2,3]. Spoilage of food by microorganisms is probably responsible for a quarter of the food waste around the world, leading to significant economic losses and underscoring food processing environments as a major concern [4,5]. Moreover, foodborne diseases are the cause of several hundred thousand deaths worldwide every year [6,7]. Reducing food microbial contamination is a continuing battle for the primary food-processing sector, especially with meat products since they provide high nutrient availability for microorganisms and are known vectors of foodborne diseases [4,8].

The quality and safety attributes of final meat products are highly dependent on the raw materials and on their processing, storage conditions, and production environments [9,10]. Indeed, it has been shown that the production environment microbiota—in addition to being potentially involved in the spread of antimicrobial resistance genes [11] and in the alteration of the integrity of processing surfaces—may pose a threat to the attributes of meat [12,13]. Stellato et al. proposed the hypothesis that an equilibrium between the food and the production environment is established since the microbiota found on the surfaces and tools used during processing are often found on the meat [14,15]. Along this vein, current food production sanitary measures mainly rely on low contamination of the production environment while little attention has been paid, as of yet, to the structure of the bacteria communities and the transmission routes of microorganisms [4,16]. Recently, Stellato et al. proposed that since the microbiota of a site is under the influence of specific characteristics such as the type of surface involved, the environmental conditions, and the processes to which the food is subjected, it can be expected that each site will present a specific microbiota [17]. A few studies using diversity analysis found this not to be the case, with environmental processing surface-samples’ clustering according to sampling time [17] and not according to a specific sampling zone [15,17]. These conflicting results highlight the need for studies that address the presence of microenvironments in the food production environment by eliminating, as much as possible, the confounding factors that can influence results and make comparisons between studies difficult.

The recent introduction of high throughput sequencing technology has allowed the efficient characterization of complex and diverse microbial communities [18]. To date, only a limited number of studies have explored the general contamination flows and the microbial organization patterns of food processing environments using 16S rRNA amplicon sequencing [4,15]. The pairing of such sequencing techniques with computational methods has made it possible to infer the sources of origin of microorganisms of interest in a given environment [4]. Several bacterial source-tracking methods have been proposed to detect the origins of bacterial contamination such as Bayesian approaches and random forest algorithms (RF) [19,20].

Slaughterhouses are increasingly identified in the meat processing sector as a key site where unwanted microorganisms are found [4]. At the slaughterhouse, carcasses can be contaminated by the animal’s endogenous microbiota during processing. It has been shown that microorganisms can remain on the surface of a carcass and can survive and detach when they come into contact with surfaces and production equipment [21]. Thus, the contaminated contact surfaces can be sites of cross-contamination for the meat products circulating on them [18]. Indeed, contact surfaces such as conveyor belts have been shown to act as reservoirs for spoilage and pathogen bacteria [21]. Biofilms can settle in irregularities of conveyor belts and act as sources of contamination [22]. In natural environments, biofilms are known to be composed of multiple species [23,24]. The identity of the microorganisms constituting them as well as their interactions have been identified as contributors to the creation of local microbial ecosystems in the production environment [24,25]. Although the nature and quantity of microorganisms found on fresh raw meat at the slaughterhouse represent an initial point that can influence the safety and the spoilage patterns during further transport and storage, and can spread contamination to ready-to-eat facility environments [26,27], very little is known about the general microbial ecology of slaughterhouse production environments, especially with regard to the contamination during further processing stages such as cutting and deboning [16]. During these stages, which take place in the cutting room, the meat carcasses undergo different modifications in order to obtain parts intended to become standard meat cuts. These meat pieces will undergo their final transformation on specific production lines. Thus, a specific production line supports the passage of a specific meat cut. Recently, de Filippis et al. shed light on the significant association between beef microbiota and specific beef cuts [27]. They showed that different cuts from the same carcass can influence the microbial contamination of beef. The influence of the type of meat cut on the microbiota of contact surfaces (production lines) is yet to be established.

Using 16S rRNA sequencing and diversity analysis, the objectives of this study were: (i) to characterize the heterogeneity of the microbiota of conveyor belt contact surfaces present in the cutting room of a swine slaughterhouse in the province of Quebec, Canada, over a period a time (six visits), and to characterize the impact of the production line on the microbiota diversity of those surfaces; (ii) to identify microbial determinants associated with the visit and production line with a focus on bacterial determinants that can include known spoilage species and known foodborne pathogens; finally, (iii) to ascertain if, by using the microbiota of a given sample, it is possible to determine which visit and which production line the sample belongs to using random forest models as a predictive tool. We believe that understanding the composition of the microbiota on food processing surfaces is crucial since these distinct bacterial community dynamics are important in determining the microbial succession patterns that will occur and will influence the quality and safety of the final meat products. This knowledge could lead to more targeted cleaning and disinfecting procedures as well as to a more accurate management of the different meat cuts for further processing. Our study proposes an original approach based on diversity analysis and random forest models to evaluate, for the first time to our knowledge, the microbiota heterogeneity of the potential cross-contamination routes driven by the different production lines and visits in a cutting room of a swine slaughterhouse.

## 2. Materials and Methods

### 2.1. Facility Structure, Sampling, Total Microbiota Harvesting, DNA Extraction, Amplicon Library Preparation, and 16S rRNA Sequencing

It should be noted that this study is part of a larger project which was developed in two phases. The first phase aimed to evaluate spatial and temporal *Listeria monocytogenes* contamination on the six production lines in a swine slaughterhouse cutting room in Quebec, Canada and to identify microbial determinants of the presence or absence of the bacteria on those surfaces [28]. The current study is the second phase of that project and aims to evaluate the microbiota’s homogeneity on contact surfaces according to processing line (each line is dedicated to a specific cut of meat) and according to different visits using the samples harvested during the first phase. The sampling procedures as well as the processing of the microbiota samples have been described in detail in the work of Shedleur-Bourguignon et al., 2021 [28].

Briefly, the conveyor belt surfaces of six production lines present in the cutting room of a swine slaughterhouse with a capacity of 600 pigs per hour were sampled during midday production on six occasions between September 2017 and March 2018. Surfaces of 900 cm^2^ were sampled, first by using small brushes in order to mobilize the surface microbiota and second by collecting the biomass with sterile wipes (Innovation Diagnostics, Saint-Eustache, QC, Canada). Six production lines were present in the cutting room, and each production line supported the passage of a specific part of the carcass intended to become a standard meat piece (according to North American standards): half-carcasses, bellies, loins, Bostons, picnics, or hams. Fifty-four surface samples were taken on the main production line (CP), 48 on the belly production line (FL), 48 on the loin production line (LO), 48 on the Boston production line (BO), 48 on the picnic production line (PI), and 48 on the ham production line (FE). One experimental control sample per visit was also taken, which consisted of wipes and brushes that were taken out of their packaging in the cutting room and not allowed to come into contact with any surfaces. Specifically, upon arrival at the laboratory facilities, the heads of the brushes were cut and added to Falcon tubes containing 25 mL of a solution composed of Tris-HCL (10 mM), EDTA (10 mM), and NaCl (0.85%). The used wipes were subsequently cut in half under sterile conditions at the lab and each half wipe was added to the solution and the samples were stomached (Biomérieux Canada, QC, Canada) (the other half wipe was tested in a previous study [28]). Twenty mL was then transferred into two conical tubes of 15 mL (Sarstedt Inc, Saint-Laurent, QC, Canada). The Falcon tubes were then centrifuged (VWR, Saint-Laurent, QC, Canada), the supernatants were discarded, and the bacterial pellets were individually stored at −80 °C until DNA extraction and purification.

The two bacterial pellets belonging to the same sample were pooled. The total DNA of the combined bacterial pellets was then extracted and purified using a modified version of a phenol–chloroform protocol as previously described [28]. Final DNA concentration was measured using the Qubit 3.0 High Sensitivity range assay (Fisher Scientific, Ottawa, ON, Canada). The purity of the DNA was evaluated by using a Nanodrop ND-1000 Spectrophotometer (Thermo Scientific, Wilmington, DE, USA) and by gel electrophoresis (1% of agarose). A 291 bp segment of the V4 hypervariable region of the 16S rRNA gene was amplified using universal primers (515F_Ill and 806R_Ill; Invitrogen, Thermo Fisher Scientific, Waltham, MA, USA) [29,30]. A 30 µL PCR reaction was carried out using the Platinum SuperFi PCR Master Mix (Invitrogen, Burlington, ON, Canada) as described by Shedleur-Bourguignon et al. 2021 [28]. An artificial community (ZymoBIOMICS Microbial Community DNA Standard) (Zymo Research, Irvine, CA, USA) served as a positive control and as an indicator of sequencing quality. The theoretical composition of the artificial community based on 16S sequencing is: 18.4% *Lactobacillus*; 17.4% *Bacillus*; 15.5% *Staphylococcus*; 14.1% *Listeria*; 10.4% *Salmonella*; 10.1% *Escherichia*, 4.2% *Pseudomonas*, and 9.9% *Enterococcus*. Six experimentation controls (wipes) and five negative sequencing controls (DNA extraction and PCR steps) were integrated into the plates. The amplicons were then sent to McGill University Genome Quebec Innovation Center (Montreal, QC, Canada) for purification, barcoding, and sequencing by Illumina MiSeq 250 paired-end sequencing.

### 2.2. Sequencing Data Processing, Diversity, and Statistical Analysis

Diversity analyses were performed in order to characterize the composition and the structure of bacterial populations present on conveyor belt contact surfaces. As previously described by Shedleur-Bourguignon et al., the cleaning and the analyzing of the sequences were executed using Mothur 1.39.5 [28,31]. Alpha and beta diversity analysis were performed using RStudio 3.6.1. The lowest number of sequences in the samples was used as subsampling. For the alpha diversity, the coverage of the subsampling was measured as well as the number of operational taxonomic units (OTUs) in each sample, and their evenness was measured using the inverse Simpson and the Shannon indices. The indices were calculated by using the estimate_richness function of the phyloseq package version 1.24.2 [32]. Comparison statistics were performed between the samples from the different visits and between the different production line samples using Student *t*-tests and the Kruskal–Wallis test, both with a significance level of 0.05. For the beta diversity, the Jaccard index based on the presence/absence of OTUs and the Bray–Curtis index based on the relative abundance of OTUs were used on the same subsampling. The distance matrices were plotted using 2D nonmetric multidimensional scaling graphs (NMDS) using the ordinate function of the phyloseq package and the ggplot2 package [33], and statistical variations between the different groups (visits and production lines) were assessed using permutational analysis of molecular variance (PERMANOVA) using the ADONIS function of the vegan package [34]. The Bonferroni correction was used in order to correct the significance level in the presence of multiple comparisons. The impact of production lines on the beta diversity of the visits was taken into consideration by adding the production line factor in strata during statistical tests. The same procedure was used to remove the influence of the visits on the beta diversity of the production lines. The Multivariate Association with Linear Models method (MaAsLin version1) was used to identify OTUs that were significantly, positively, or negatively associated with each visit and with each production line in terms of relative abundance using the MaAsLin package [35]. The two variables (visit and production line) were used as a fixed effect and were put into the boost formula to consider the influence of the variables on each other. An association was considered significant at a *p*-value ≤ 0.05 and a *q*-value ≤ 0.25.

### 2.3. Evaluation of the Predictability of a Visit and a Production Line Using Random Forest Models

The models were built in order to classify the samples according to the visit during which they were collected or according to the production line from which they came based on their microbiota. The high number of covariates (OTUs) implicated in the analysis as well as the potential interactions between them guided the choice of using a nonparametric random forest approach as a predictive tool [36]. Two predictive models were built with RStudio 3.6.1 using the RandomForest and the caret packages [37,38]. For each of the two models, eight randomly chosen samples per visit and eight randomly chosen samples per production line were used as a test database to evaluate the performance of the models in order to avoid overfitting. The remaining samples were used to fit the random forest models. As each run of random forest could produce different results, one hundred random forests were performed per model to assess the robustness of the results. A median accuracy (rate of correct predictions) and an empirical 95% confidence interval were estimated to evaluate the performance for each model on the 100 iterations. Confusion matrices of the predictions were also computed on the first iteration of random forest for each model using the yardstick package [39]. The importance of each OTU in the prediction of a sample belonging to a particular production line or visit was determined to identify the most relevant OTUs. The importance was computed using the median of the decrease in accuracy when an OTU is removed from the model among the 100 replications of random forest (Liaw and al., 2002). In order to identify these OTUs, a random noise variable was introduced in the model. OTUs with a higher importance than the random noise in 95% of the replications of the random forest were considered as important in the prediction.

## 3. Results

### 3.1. 16S rRNA Gene Amplicon Sequencing

The V4 region of the 16S rRNA gene was sequenced from 310 samples: 294 conveyor belt surface samples were collected on six production lines over six visits, six experimentation controls (one per visit), five negative sequencing controls (PCR and sequencing negative controls), and five DNA positive controls (ZymoBIOMICS Microbial Community DNA Standard). A total of 7,198,363 sequences with an average of 23,437 sequences per sample were obtained after read processing and were grouped into 33 phyla, 84 classes, 152 orders, 313 families, and 952 operational taxonomic units (OTUs). The experimentation controls and the negative sequencing controls contained an average of 16,536 sequences and 7692 sequences, respectively, and no band was visible on gel electrophoresis for these two types of controls prior to sequencing. The ZymoBIOMICS Microbial Community DNA Standard positive controls were composed of the eight bacterial genera expected and showed an average of 17,733 sequences. The average composition of the positive samples based on 16S sequencing was: 16.2% *Salmonella*; 10.0% *Escherichia*/*Shigella*; 13.8% *Bacillus*; 13.2% *Staphylococcus*; 17.7% *Lactobacillus*; 11.5% *Listeria*; 9.5% *Pseudomonas*; and 4.4% *Enterococcus*.

### 3.2. Production Line Surface Microbiota Description

In order to avoid the description of the sporadic presence of bacterial genera and to instead focus the analyses on results of higher biological value, only the bacterial genera (OTUs) with a relative abundance equal to or greater than 5% in at least five samples from a visit or a production line were compiled in Table 1 and Table 2. Therefore, the 15 most abundant OTUs for all visits combined were from the most abundant to least abundant: *Fusobacterium* (13.5%); *Trueperella* (12.9%); *Peptoniphilus* (12.7%); *Acinetobacter* (11.9%); *Pseudomonas* (11.1%); *Bacteroides*_unclassified (10.6%); *Rothia* (9.9%); *Aerococcaceae*_unclassified (9.7%); *Porphyromonas* (9.7%); *Clostridium_sensu_stricto* (9.6%); *Enhydrobacter* (9.5%); *Staphylococcus* (9.3%); *Macrococcus* (8.7%); *Psychrobacter* (8.4%); and *Halomonas* (8.3%). The 15 most abundant OTUs for all the production lines combined were: *Fusobacterium* (18.8%); *Rothia* (15.6%); *Trueperella* (14.7%); *Peptoniphilus* (14.6%); *Acinetobacter* (13.8%); *Staphylococcus* (12.6%); *Enhydrobacter* (12.1%); *Aerococcaceae*_unclassified (11.3%); *Peptostreptococcus* (10.7%); *Bacteroides*_unclassified (10.5%); *Macrococcus* (10.3%); *Pseudomonas* (10.1%); *Porphyromonas* (10.1%); *Clostridium*_sensu_stricto (9.6%), and *Psychrobacter* (7.6%).

### 3.3. Microbiota Diversity of Conveyor Belt Surfaces Based on Visit and on Production Line

#### 3.3.1. Alpha Diversity

Alpha diversity analyses were performed in order to describe the bacterial richness and distribution within samples depending on visit and production line using the Observed index, the Shannon Evenness index, and the Inverse Simpson index. Alpha diversity measures of visit samples and production line samples are shown in Table 3 and Table 4. Student’s *t*-test revealed differences in alpha diversity between several pairs of visits and between multiple pairs of production lines using the three indices. Kruskal–Wallis tests showed significant differences between the visits with the Shannon Evenness and Inverse Simpson indices as well as differences between the production lines with the three indices.

#### 3.3.2. Beta Diversity

**Figure 1:**
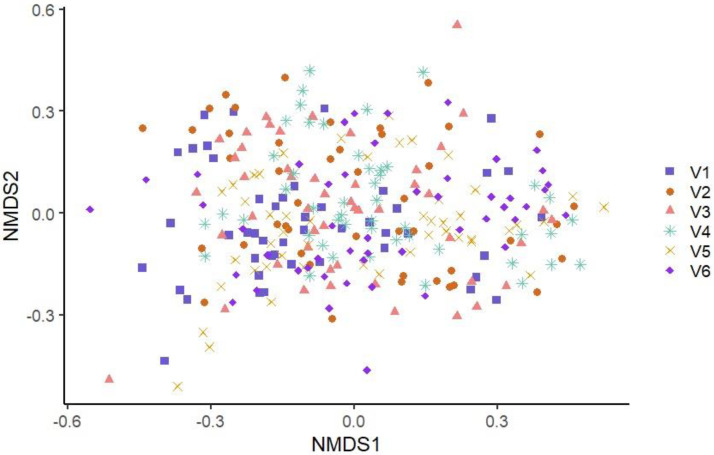
Non-Metric Multidimensional Scaling Graph (NMDS) of the microbiota structures of the six visit samples using Bray–Curtis index. Each point represents a sample, and each visit is represented by the combination of a symbol and a color. Blue square: Visit one; Orange circle: Visit two; Pink triangle: Visit three; Turquoise star: Visit four; Yellow x: Visit five; Purple diamond: Visit six.

**Figure 2:**
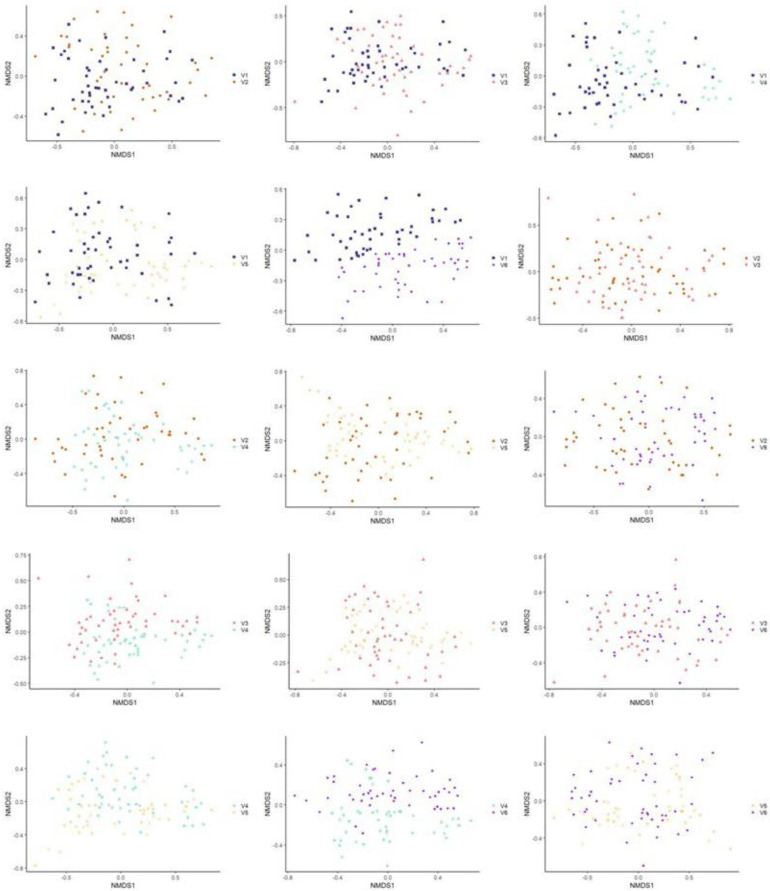
Non-Metric Multidimensional Scaling Graphs (NMDS) using Bray–Curtis index illustrating sample microbiome beta-diversity according to visit and paired two by two. Blue square: Visit one; Orange circle: Visit two; Pink triangle: Visit three; Turquoise star: Visit four; Yellow x: Visit five; Purple diamond: Visit six.

**Figure 3:**
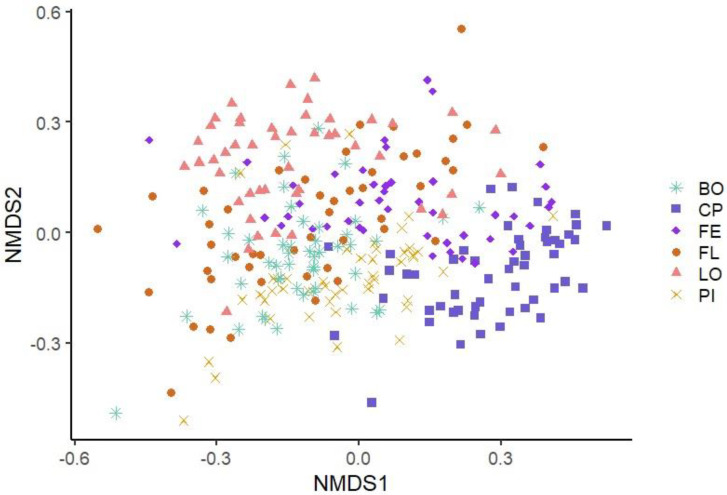
Non-Metric Multidimensional Scaling Graph (NMDS) of the microbiota structures of the six production line samples using Bray–Curtis index. Each point represents a sample, and each production line is represented by the combination of a symbol and a color. Turquoise star: Boston production line (BO); Blue square: Main production line (CP); Purple diamond: Ham production line (FE); Orange circle: Belly production line (FL); Pink triangle: Loin production line (LO); Yellow x: Picnic production line (PI).

**Figure 4:**
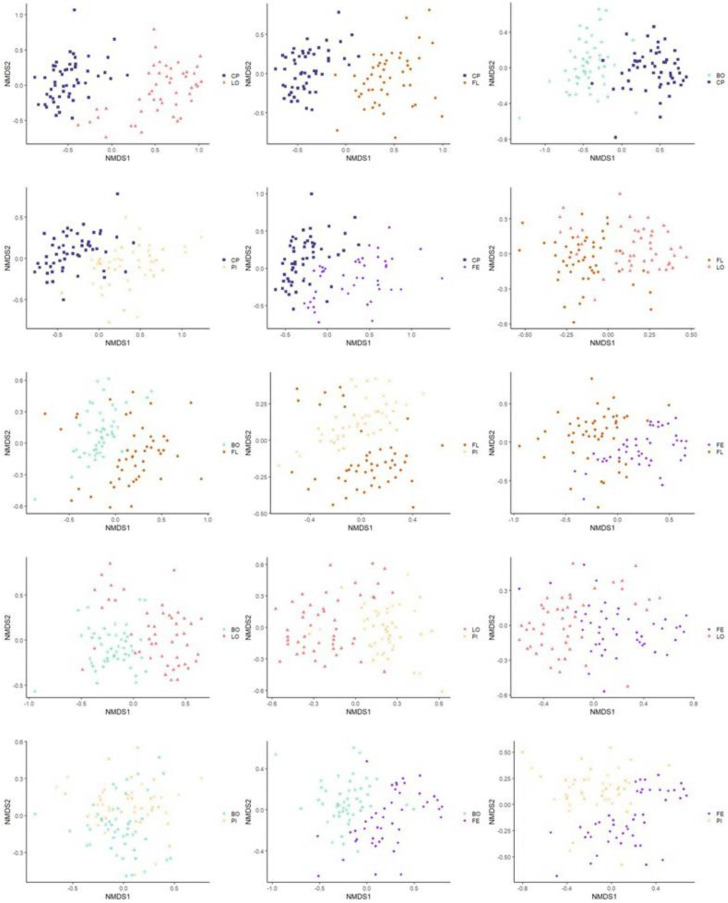
Non-Metric Multidimensional Scaling Graphs (NMDS) using Bray–Curtis index illustrating sample microbiome beta-diversity according to production line and paired two by two. Turquoise star: Boston production line (BO); Blue square: Main production line (CP); Purple diamond: Ham production line (FE); Orange circle: Belly production line (FL); Pink triangle: Loin production line (LO); Yellow x: Picnic production line (PI).

The similarity of the microbiota’s structure was compared at the OTU level between the samples according to the different visits using the Bray–Curtis index and the Jaccard index. The similarity of the microbiota’s structure was also compared between the samples according to the different production lines using the same indices. Statistically significant differences were found between all pairs of visits (*p* ˂ 0.001) with both indices. Figure 1—which illustrates the structure of the microbiota between samples belonging to the different visits using the Bray–Curtis index—does not show apparent clustering between samples belonging to the same visit (see Figure 1). However, when each visit was plotted in two-by-two NMDS plots, more important differences emerged between some pairs of visits: V1 and V3, V1 and V4, V1 and V5, V3 and V4, and V4 and V5 (see Figure 2). Statistically significant differences were also found between all pairs of production lines (*p* ˂ 0.001) with the Jaccard and the Bray–Curtis indices. These differences in bacterial community composition of the different production lines can be clearly visualized in an NMDS graph using the Bray–Curtis index (see Figure 3). Figure 3 shows a clustering between samples from the same production line. This clustering is even more apparent when the different production lines were plotted two-by-two (see Figure 4). Similar distance matrix NMDS plots were obtained using the Jaccard index (see Figures S1–S4 (Supplementary Materials)).

#### 3.3.3. Multivariate Association with Linear Model Analysis

Multivariate association with linear model analysis (MaAsLin 1) was assessed to identify OTUs positively or negatively associated with the different visits and the different production lines. The number of total OTUs positively and negatively associated with the different visits in terms of relative abundance as well as a summary of associated OTUs of interest (bacterial genera, which can include known spoilage species and known foodborne pathogens species) are detailed in Table 5. Production line associations from the MaAsLin test are presented in Table 6. The information is presented in the same way as the MaAsLin visit data. The complete list of the taxonomic attribution of OTUs associated with the various visits and production lines is available in the Supplementary Materials (see Tables S1–S4).

### 3.4. Random Forest

**Figure 5:**
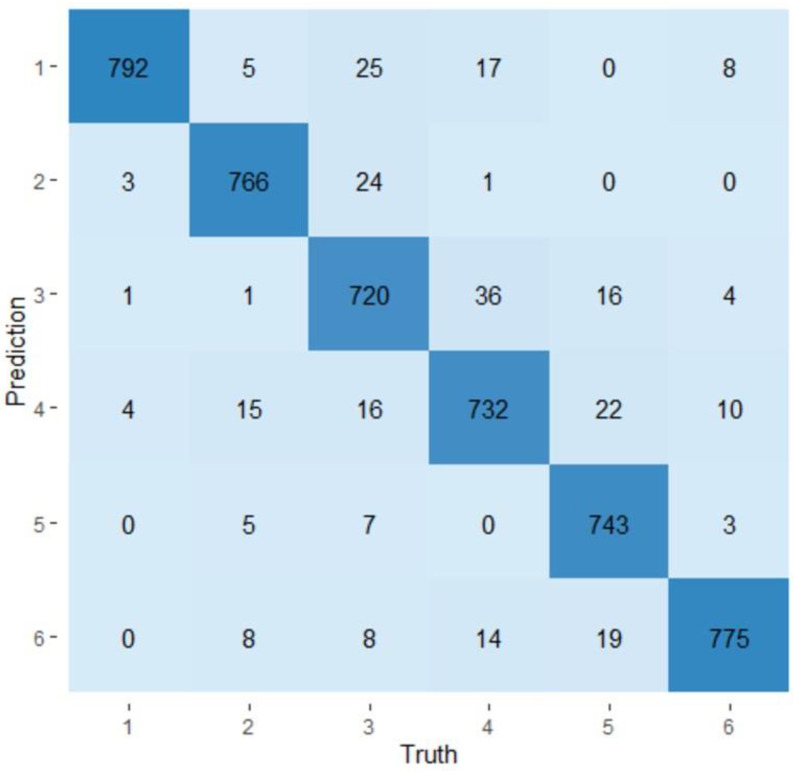
Confusion matrix of the visit model. The *x*-axis line indicates the identity of the six visits and the *y*-axis line shows the prediction of a sample visit based on its microbiota. 1: Visit one; 2: Visit two; 3: Visit three; 4: Visit four; 5: Visit five; 6: Visit six.

**Figure 6:**
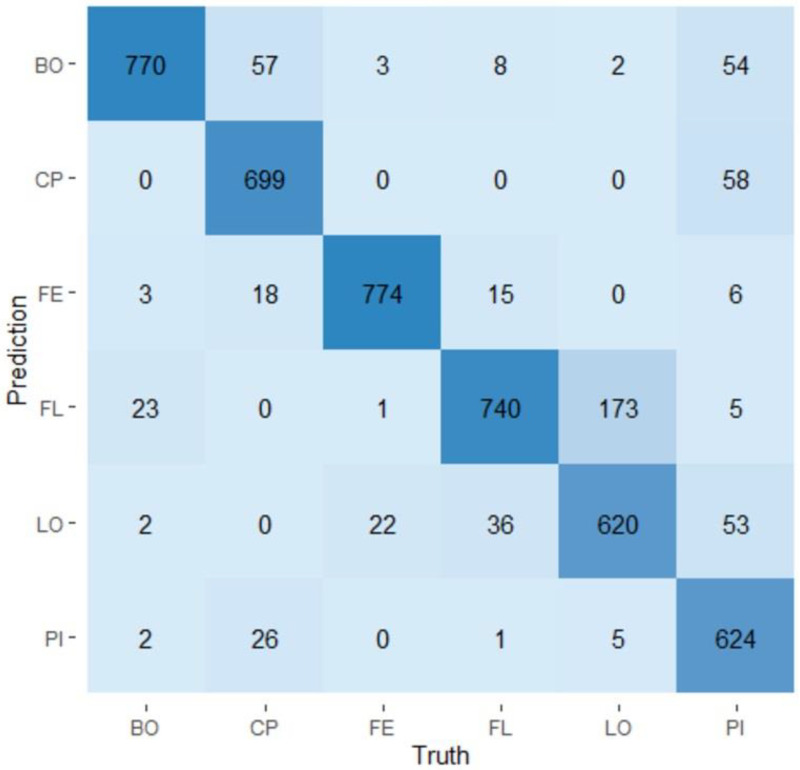
Confusion matrix of the production line model. The *x*-axis line indicates the identity of the six production lines and the *y*-axis line shows the prediction of a sample production line based on its microbiota. BO: Boston production line; CP: main production line; FE: Ham production line; FL: Belly production line; LO: Loin production line; PI: Picnic production line.

The two models based on random forests were used to classify the samples according to the visit during which they were collected or according to the production line from which they came based on their microbiota. The confusion matrices obtained for the visit model and for the production line model are presented in Figure 5 and Figure 6, respectively. The visit model had an average prediction rate of 94.3% (89.6–100.0%). Visit one showed the best prediction rate (99.0%) and visit three showed the lowest rate of prediction (90.0%). Visits two, four, five, and six showed prediction rates of 95.8%, 91.5%, 92.9%, and 96.9%, respectively. The production line model has an average production rate of 88.1% (81.3–93.9%). The ham production line showed the best prediction rate (96.8%), while the loin production line showed the lowest prediction rate (77.5%). The main production line, the belly production line, the Boston production line, and the picnic production line, respectively, showed prediction rates of 87.4%, 92.5%, 96.3%, and 78.0%. The importance of variables (OTUs) in the prediction of a visit or a production line was evaluated. Among the 952 OTUs determined in the study, only a small proportion of them—143 and 182—were considered important to predict the visits and the production lines, respectively. The complete lists of these OTUs can be seen in Tables S5 and S6.

## 4. Discussion

In our study, a total of 294 samples of production line surfaces associated with the circulation of different meat cuts were collected in the cutting room of a swine slaughterhouse. The microbiota of the samples was analyzed by 16S rRNA amplicon sequencing in order to evaluate the impact of visit and production line on the diversity and structure of bacterial communities found on contact surfaces. The majority of the bacterial sequences collected in our study belonged to the phyla *Proteobacteria* (36.4%), *Firmicutes* (35.9%), *Fusobacteria* (31.4%), *Actinobacteria* (30.0%), and *Bacteroidetes* (27.2%). The dominance of these phyla in pig slaughterhouse environments has been reported by previous studies, with the exception of the *Fusobacteria* phylum [40,41]. The *Fusobacteria* phylum is known to be a normal constituent of the oropharyngeal and gastrointestinal microbiota of pigs [42]. A recent study by Wylensek et al. revealed pig-specific species within the *Fusobacterium* genus [43]. Thus, bacteria belonging to this phylum could have been transferred from the gastrointestinal tract of pigs to the carcasses during evisceration and then transferred to surfaces in contact with the meat [21,44]. The dominance of the phylum *Fusobacteria* can potentially be explained by the specific context of our study since our study was interested in the microbiota of conveyer belt surfaces in a cutting room during production while most of the available studies focused on multiple slaughterhouse compartments/rooms [12]. Our results showed that the *Fusobacteria* phylum had a significantly higher relative abundance during the fourth and the sixth visits and that the phylum was important for the identification of the visit and production line in the random forest models.

Several genera found among the 15 most abundant bacteria across all visits and production lines combined have been characterized in the literature as having phenotypes allowing them to counteract the stress conditions encountered in the production environment. Indeed, the dominant presence of psychotropic genera such as *Pseudomonas*, *Acinetobacter*, and *Psychrobacter* was expected due to the refrigeration temperatures maintained in the cutting room [12]. A study by Botta et al. correlated the greater abundance and persistence of these three genera in secondary processing rooms with the lowest temperatures of these environments [26]. In addition, several of the most abundant phyla in our study, such as *Staphylococcus*, are known to be able to form biofilms. Biofilms can confer a greater resistance to disinfection products to bacteria but also to shear forces produced by moving parts in production [45]. A recurrent introduction of microorganisms by the carcasses following the contact of meat products with processing surfaces in the cutting room can also not be excluded. Several bacterial genera with potentially pathogenic species and/or potential spoilage species were among the OTUs with a relative abundance equal to or greater than five percent in at least five samples from a visit or production line. The presence of these OTUs on fresh pork meat have been previously described [10]. However, the presence of the bacterial genus *Clostridium* among the 15 most abundant OTUs during visits and on production lines and its strong presence in the cutting room was not expected. Even though the *Clostridium* genus is not known to be a problem in the environmental processing environment of swine slaughterhouses regarding its potential pathogenicity for humans, the high representation of the genus *Clostridium* in our study suggests that the presence of this potential foodborne pathogen may be underestimated.

Variability in the mean relative abundance of frequent bacterial genera (OTUs) was identified. Indeed, several genera showed a higher relative abundance during certain visits or on certain production lines. For instance, the genus *Staphylococcus* showed a higher relative abundance during the third visit. The genera *Peptostreptococcus* (V2) and *Macrococcus* (V4) are also examples of bacteria overrepresented during a single visit. In addition, our study showed statistical differences in terms of alpha diversity between several pairs of visits with the three indices. Moreover, statistically significant differences of beta diversity were found between all pairs of visits, indicating that the structures of major and minor bacterial populations are affected by the day of production. Put together, these results, as expected, suggest that the number of different bacterial genera and the uniformity of their distribution on surfaces are affected by the day of production. Indeed, in a different context, Stellato et al. investigated the bacterial biogeographical patterns on surfaces and tools in a hospital cooking center and showed that, based on the composition of the microbiota, samples from food production environments grouped according to sampling time (two months separated the two samplings) [17]. As the slaughter process is thought to present very little variation, it is possible to hypothesize that the differences in the mean relative abundance as well as in alpha and beta diversity are mainly supported by the arrival of different inputs (carcasses) in the cutting room, by the staff working during these different days, and by differences in the application of washing and disinfection measures. However, a study by Braley et al., conducted in the same facility as our study, revealed no significant difference of microbiota between pig carcass batches [46]. The fact that the study conducted by Braley et al. was realized during a short period of time (one day) and the sampling in our study took place at monthly intervals could explain these variations in the impact of time. Another study by Lim et al. showed that depending on the season, changes in the abundance of certain bacterial genera could be observed on food-contact and non-food contact surfaces in a foodservice facility [18]. The impact of season on the microbiota composition of fresh beef meat was also highlighted in a study by Hwang et al. [47]. As our sampling took place over six months (one sampling each month) and covered three seasons (summer, autumn, and winter, which are notably distinct in Canada), it is possible that conditions specific to the seasons have influenced the quantity and diversity of the microorganisms harvested. It should be noted that the impact of the visits on the diversity of the processing surfaces microbiotas associated with production line was taken into consideration in the present study.

Variability in the mean relative abundance of bacterial genera (OTUs) was also identified in relation to the production line. Indeed, the genera *Trueperella* (FL), *Pseudomonas* (FE), *Acinetobacter* (LO), *Rothia* (LO), and *Staphylococcus* (LO) presented a higher mean relative abundance on certain production lines. In the same perspective, a study by Biasino et al. mapped the distribution of microbiological contamination of pig carcasses [44]. They showed significant differences, using a culture-dependent methodology, in the contamination level of the different carcass areas regarding the total aerobic bacteria *Enterobacteriaceae* and *Salmonella*. De Filippis et al., for their part, have highlighted a significant association between beef microbiota and specific beef cuts using 16S rRNA amplicon sequencing [27]. The results of these two studies that highlight the influence of different meat cuts on the microbial contamination of meat are in line with our observations but leave unanswered the link between the microbiota of the production surfaces and the pieces of meat they support.

Our study showed statistical differences in terms of alpha diversity between several pairs of production lines. The highest alpha diversity measures have been associated with the CP and the FE production lines. The BO production line showed the lowest alpha diversity values while the FL, LO, and PI production lines showed variable levels of diversity depending on the index used. In addition, statistically significant differences of beta diversity were found between all pairs of production lines. This is the first time to our knowledge that a study has revealed the presence of bacterial microbiotas distinct in number, uniformity, and structure between production lines in a cutting room of a slaughterhouse. As the only difference that exists between the conveyors associated with the different production lines is the type of meat cut circulating on them, our study represents a first step in the exploration of the influence of meat cut on surface contact microbiota. However, these results were obtained in a single slaughterhouse, therefore it would be interesting and relevant to study whether these observations are present in other cutting rooms of pig slaughterhouses. These results concur with several studies which have highlighted the presence of different microbiotas in the food production environment. Zwirzitz et al. applied the software SourceTracker on full-length 16S rRNA gene sequencing data from a swine slaughterhouse and revealed that contact surfaces such as the polishing tunnel and a railing used for the classification of the carcasses strongly contributed to the composition of the microbiota of meat samples [4]. The study showed that many bacterial genera were unique to specific sites in a swine slaughterhouse, indicating that specific microorganisms occupy environmental niches in the facility. The authors suggested that the spatial distribution of the microorganisms is the result of specific transmission routes within the facility. Botta et al. conducted a study in three red-meat slaughterhouses and revealed significant differences in the meat processing-plant surface microbiota between the environments (the three different plants), the temporal phases (before or after cleaning and sanitizing), and between the type of room (deboning rooms and processing room). Although these studies have explored the general contamination flows and the microbial organization patterns [1,4] of food processing environments, none of them focused on a single compartment/room in a food plant.

Multivariate association with linear model analysis (MaAsLin) was assessed to identify OTUs positively or negatively associated with the different visits and the different production lines. Among the determinants positively or negatively associated with the different visits, 12 have been previously identified as possibly containing known spoilage species and known foodborne pathogen species: *Acinetobacter*, *Flavobacterium*, *Pseudomonas*, *Psychrobacter*, *Lactobacillus*, *Lactococcus*, *Streptococcus*, *Brochothrix*, *Carnobacterium*, *Clostridium, Enterococcus*, and *Aerococcus*. The identification of visits during which these microbial genera showed significantly higher relative abundance on contact surfaces raises the question as to whether there are differences in the relative abundance of these bacterial genera on the carcasses. Indeed, it has been shown that microorganisms can remain on the surface of the carcass, survive, and detach when they come in contact with surfaces and production equipment [21]. Six of those bacterial genera currently recognized as spoilage determinants were lactic acid bacteria genera (LAB): *Lactobacillus*, *Lactococcus*, *Carnobacterium, Aerococcus, Streptococcus*, and *Enterococcus*. LAB are recognized as important competitors of other spoilage microorganisms and have been reported to be associated with the souring, formation of slime, bone taint, and greening of refrigerated fresh raw meat [10,48,49]. In addition to the lactic acid bacteria, the genus *Brochothrix* (Otu00245), another Gram-positive bacterium, emerged as a determinant in the MaAsLin tests. The species *B. thermosphacta* is known as an important spoiler of various food matrixes, including the formation of slime on pork meat [10,48]. A study by Hultman and al. suggested that refrigerated food-processing environments provide conditions for the persistence of *Brochothrix* spp and LAB and that meat-contact surfaces could act as reservoirs for those bacteria [15]. The *Acinetobacter* and *Pseudomonas* genera, for their part, are considered as important sources of meat spoilage products in aerobic conditions [50], while several species of the genus *Clostridium* have been identified as causative agents for defects of meat in anaerobic conditions [49].

Regarding the production lines, 12 have been previously identified as common potential meat spoilage bacterial genera. In our study, several LAB (*Lactococcus*, *Lactobacillus, Carnobacterium*, and *Enterococcus*) showed a positive association in terms of relative abundance with the CP, LO, and FE production lines, suggesting predicted locations of LAB reservoirs on production lines. The genus *Clostridium* was the only genus identified as a determinant of five production lines (CP, FL, LO, PI, and FE). However, it is interesting to note that the distribution of the OTUs (OTUs 00016, 00059, 00136, 00203, 00307, and 00441) associated with this bacterial genus is differentially organized. For example, the OTU 00203 and the OTUs 00307 and 00441 were found only on the PI and CP production lines, respectively. This suggests that particular *Clostridium* species would be specifically associated with certain production lines. Thus, since only a fraction of the *Clostridium* species is identified as a causative agent for defects in vacuum-packed meat [49], precisely identifying the species associated with the different production lines could help identify the cuts of meat best-suited for this type of packaging. Except for the genus *Clostridium*, no OTU identified as potential spoilage bacteria showed a positive association with the FL production line. On the contrary, four bacterial genera associated with meat spoilage showed a negative association with the FL production line: *Psychrobacter*, *Moraxella*, *Acinetobacter*, and *Pseudomonas* [14,51]. The PI production line also showed a few positive associations (*Clostridium* and *Psychrobacter* genera) but also three negative associations (*Acinetobacter*, *Pseudomonas*, and *Salmonella* genera). While *Psychrobacter* and *Moraxella* possess a low spoilage potential due to their lack of several important biochemical attributes such as proteolysis, *Pseudomonas*, the species *P. fluorescens*, *P. putida*, and *P. fragi*, in particular contribute to the spoilage of raw meat to a large extent [52]. No association was found with the BO production line. These results suggest, in the context of our study, that the BO, the FL, and the PI production lines are the least-concerned production lines in terms of relative abundance regarding bacteria capable of deteriorating meat quality.

Four bacterial genera potentially pathogenic to humans were found among the bacterial determinants positively or negatively associated with the different visits. The potential pathogenic genus *Campylobacter*, of which species *C. coli* frequently contaminates pigs and consequently food of porcine origin, was identified as a microbial determinant of visits five and six [53]. The genus *Listeria*, of which the species *L. monocytogenes* has been linked to several outbreaks associated with the consumption of pork products, was associated with the second visit [54,55]. Based on these results, the introduction of these two potential pathogens seems to have been sporadic. It is therefore possible to hypothesize that the inputs (carcasses) introduced into the cutting room on these two dates had a higher relative abundance of *Campylobacter* or *Listeria* than the inputs from the other visits and could therefore have transferred these pathogens in higher concentrations to the contact surfaces. This observation is reinforced by the fact that the OTU associated with the genus *Listeria* was unique to visit two.

The *Campylobacter* genus also showed positive associations with the CP and the FE production lines in the same way as the genus *Escherichia/Shigella*, an indicator of fecal contamination, with the FL and the FE production lines [56]. The *Salmonella* genus, a major concern of pork meat-product contamination, showed a negative association with the PI production line [57,58]. The results of our study are partially in accordance with a study by Biasino et al. [44], which showed that the ventral and anterior areas (foreleg, head, sternum, and throat) were the most contaminated parts of pig carcasses by *Enterobacteriaceae* and *Salmonella.* These results are consistent with our study, which showed a lower relative abundance of the genus *Salmonella* on the PI production line. However, for *E. coli*, our study showed a higher relative abundance of the *Escherichia/Shigella* genera on the FE production line [44]. This divergence in results can be explained by differences between the production facilities sampled, such as the evisceration techniques and the cleaning and disinfection procedures, by the use of different detection methods, and also by the fact that the Biasino et al. study was conducted on carcass parts while our study was conducted on surfaces in contact with these carcass parts [44]. Unfortunately, few studies have examined the transmission of *Campylobacter* in the pork industry, which does not allow the comparison of our results. The *Staphylococcus* genus showed a unique positive association with the LO production line. This bacterial genus is a potential opportunistic pathogen of which pigs and humans are potential hosts [59]. As no association was found with any visit, the hypothesis of an introduction via workers on this particular production line is probable. The presence on several conveyors of the genus *Clostridium*, within which the *C. perfingens* species represents a risk to humans, has been discussed above. The identification (using the MaAsLin test) of microbial determinants positively or negatively associated with the different production lines shows for the first time to our knowledge the existence of significant differences in terms of relative abundance in the organization of microbial communities on the production lines associated with specific meat cuts at the slaughterhouse.

Having identified bacterial determinants associated with the different visits and the different production lines, two predictive models were built based on random forests as proof of concept in order to establish, through the predictive character of these models, the existence of distinct microbiotas in a cutting room of a slaughterhouse. Based on the microbiota of a sample, our models were able to predict its visit number at a rate of 94% and which production line it came from at a rate of 88%. The proof of concept provided by the creation of our predictive models suggests their usage on a larger scale and their potential integration into larger surveillance systems based on blockchain technology. Using these models could be helpful in predicting the origin of a problematic sample quickly and efficiently. The predictive value of surface provenance based on a sample of the microbiota of a piece of meat remains to be explored.

We believe that the results of our study, which identified for the first time to our knowledge the presence of distinct microbiotas on contact surfaces related to the circulation of specific meat cuts, represent a crucial step in the understanding of the microbial ecology of a slaughterhouse. The distinct bacterial community dynamics which take place on the production lines in a cutting room are important in determining the microbial succession pattern that will occur and ultimately influence the quality and safety of the final meat products. Therefore, this study is a step forward in the prevention, surveillance, and control of the microbial contamination of meat products.

## Figures and Tables

**Table 1:** Bacterial genera with an average relative abundance equal to or greater than 5% according to the six visits independent of production line.

Genus	V1		V2		V3		V4		V5		V6	
	%	Nbr	%	Nbr	%	Nbr	%	Nbr	%	Nbr	%	Nbr
*Fusobacterium*	16.66	42	10.47	24	14.39	39	12.72	21	12.97	34	13.94	34
*Trueperella*	11.27	23	13.17	20	13.62	26	11.98	17	13.50	27	13.82	31
*Pseudomonas*	10.31	17	12.45	23	11.1	21	13.49	32	8.88	24	10.15	20
*Acinetobacter*	14.32	22	11.56	20	12.51	22	12.22	34	8.80	20	12.19	20
*Peptoniphilus*	13.32	15	15.29	9	13.01	9	10.36	5	13.50	11	10.93	31
*Rothia*	11.19	13	12.8	17	10.12	5	7.43	5	8.56	6	9.36	5
*Enhydrobacter*	10.96	8	10.48	9	15.92	7						
*Staphylococcus*							23.06	9	10.34	7		
*Bacteroides*_unclassified	12.12	24	9.88	11	7.54	11	12.53	5	11.45	26	9.79	12
*Aerococcaceae*_unclassified					6.77	6	12.45	12	12.58	16	13.00	12
*Psychrobacter*			7.07	8	9.12	5	10.40	10				
*Porphyromonas*	11.35	11	9.35	8	10.84	19	7.22	7	12.45	15	6.78	5
*Parvimonas*					5.9	5					7.57	17
*Clostridium_sensu_stricto*			9.34	5					9.64	6	9.58	8
*Peptostreptococcus*			16.69	6								
*Macrococcus*	8.98	8					18.58	9				
*Chryseobacterium*			6.74	6			7.25	9				
*Epilithonimonas*	11.6	7										
*Prevotella*											7.43	10
*Clostridiaceae*_1_unclassified			6.64	5								

Only the bacterial genera (OTUs) with a relative abundance equal to or greater than 5% in at least five samples from a visit were compiled. V1: Visit one; V2: Visit two; V3: Visit three; V4: Visit four; V5: Visit five; V6: Visit six. %: Average relative abundance of OTU in samples present at 5% or more. Nbr: Number of samples in which OTU is present at 5% or more. OTUs with an increase in mean relative abundance of at least 5% compared to other visits are identified in green.

**Table 2:** Bacterial genera with an average relative abundance equal to or greater than 5% according to the six production lines and independent of visit.

Genus	CP		FL		LO		BO		PI		FE	
	%	Nbr	%	Nbr	%	Nbr	%	Nbr	%	Nbr	%	Nbr
*Fusobacterium*	14.63	28	24.46	33	17.24	21	16.32	44	23.48	35	16.36	33
*Trueperella*	15.39	19	24.28	36	11.35	6	12.17	41	14.51	27	10.49	15
*Pseudomonas*	9.17	32	7.68	10	8.03	22	9.24	22	9.34	13	17.04	38
*Acinetobacter*	10.57	32	10.34	13	21.39	22	14.75	24	12.3	17	13.5	30
*Peptoniphilus*	10.19	5	16.58	20	10.46	8	15.24	15	19.99	15	14.97	17
*Rothia*	10.27	28			34.35	7			10.98	11	6.92	5
*Enhydrobacter*					14.52	9	15.74	5	14	5	10.82	5
*Staphylococcus*					16.34	15						
*Bacteroides*_unclassified	8.13	12	10.26	20	9.89	5	12.23	20	15.53	27	6.65	5
*Aerococcaceae*_unclassified	10.38	16	17.51	15	14.87	10			8.07	8		
*Psychrobacter*	10.23	16									5.73	8
*Porphyromonas*	8.58	14			14.92	6	9.37	14	8.73	10	10.12	17
*Parvimonas*			7.06	6			7.93	6			6.43	11
*Clostridium_sensu_stricto*	10.46	18									9.26	5
*Peptostreptococcus*									13.82	8		
*Macrococcus*					11.91	24						
*Streptococcus*	8.02	5										
*Chryseobacterium*	7.87	9										
*Epilithonimonas*											11.43	8
*Rhodopseudomonas*	5.98	9										
*Prevotella*											7.94	6
*Clostridiaceae*_1_unclassified					6.62	5						

Only the bacterial genera (OTUs) with a relative abundance equal to or greater than 5% in at least five samples from a production line were compiled. CP: Main production line; FL: Belly production line; LO: Loin production line; BO: Boston production line; PI: Picnic production line; FE: Ham production line. %: Average relative abundance of OTU in samples present at 5% or more. Nbr: Number of samples in which OTU is present at 5% or more. OTUs with an increase in mean relative abundance of at least 5% on one production line over any other lines are shown in green.

**Table 3:** Comparison of the alpha-diversity indices of the microbiota samples from the six visits.

	Observed	Shannon	Inv. Simpson
V1	193 *^V3, V4, V5, V6^	2.97 *^V2, V3, V4, V5^	9.85 *^V2, V4, V5^
V2	218	3.28 *^V1, V6^	13.64 *^V1, V6^
V3	221 *^V1^	3.16 *^V1, V4, V5^	11.17 *^V4, V5^
V4	239 *^V1^	3.39 *^V1, V3, V6^	14.51 *^V1, V3, V6^
V5	245 *^V1^	3.47 *^V1, V3, V6^	16.5 *^V1, V3, V6^
V6	226 *^V1^	3.07 *^V2, V4, V5^	10.35 *^V2, V4, V5^
All visits *p*-values	0.0838	0.0008 **	0.0003 **

Diversity indices were calculated with 1000 iterations based on 10,087 sequences. Each line in the graph corresponds to the alpha diversity measures associated with a visit. Pairs of visits showing a significant difference of alpha diversity according to Student’s *t*-tests are indicated with superscript after the symbol * at the appropriate line. For example, for the Observed Index, visit one is significantly different from visits three, four, five, and six. V1: Visit one; V2: Visit two; V3: Visit three; V4: Visit four; V5: Visit five; V6: Visit 6. ** Significant differences with the Kruskal-Wallis test.

**Table 4:** Comparison of alpha-diversity indices of the microbiota samples from the six production lines.

	Observed	Shannon	Inv. Simpson
CP	372 *^LO, FL, BO, PI, FE^	3.73 *^LO, FL, BO, PI, FE^	18.5 *^LO, FL, BO, PI, FE^
LO	169 *^CP, PI, FE^	3.22 *^CP, FL, BO^	11.87 *^CP, FL^
FL	180 *^CP, FE^	2.95 *^CP, LO, FE^	9.18 *^CP, LO, FE^
BO	167 *^CP, PI, FE^	3.03 *^CP, LO, FE^	10.46 *^CP, FE^
PI	196 *^CP, LO, BO, FE^	3.06 *^CP, FE^	11.51 *^CP^
FE	237 *^CP, LO, FL, BO, PI^	3.29 *^CP, FL, BO, PI^	13.52 *^CP, FL, BO^
All production lines *p*-values	2.2 × 10^−16^ **	4.64 × 10^−10^ **	1.32 × 10^−8^ **

Diversity indices were calculated with 1000 iterations based on 10,087 sequences. Each line in the graph corresponds to the alpha diversity measures associated with a production line. Pairs of production lines showing a significant difference of alpha diversity according to Student’s *t*-tests are indicated with superscript after the symbol * at the appropriate line. For example, for the observed index, the FL production line is significantly different from the CP and FE production lines. CP: Main production line; FL: Belly production line; LO: Loin production line; BO: Boston production line; PI: Picnic production line; FE: Ham production line. ** Significant differences with the Kruskal-Wallis test.

**Table 5:** Relevant bacteria (i.e., commonly known bacterial genera that can include potential spoilage species or potential foodborne pathogen species) extracted from the list of OTUs identified using the MaAsLin test and associated with the different visits.

	Positive Associations			Negative Bacterial Associations		
	Total	Unique	Relevant Bacterial Genera	Total	Unique	Relevant Bacterial Genera
V1	0	0		0	0	
V2	14	4	***Listeria_80__Otu00067***	20	1	***Escherichia_Shigella_Otu00046***
						*Brochothrix_Otu00245*
						*Streptococcus_Otu00029*
V3	39	10	*Acinetobacter_Otu00084*	20	2	*Lactococcus_Otu00132*
			*Flavobacterium_Otu00354*			*Streptococcus_Otu00029*
			*Pseudomonas_Otu00173*			
			*Psychrobacter_Otu00047*			
V4	39	11	*Acinetobacter_Otu00084*	23	1	***Escherichia_Shigella_Otu00046***
			*Enterococcus_77__Otu00071*			*Aerococcus_Otu00053*
			*Flavobacterium_Otu00138*			*Lactococcus_Otu00132*
			*Lactobacillus_Otu00092*			
			*Psychrobacter_Otu00047*			
			*Streptococcus*_Otu00024			
V5	49	12	***Campylobacter_Otu00197***	22	1	***Escherichia_Shigella_Otu00046***
			*Brochothrix_Otu00245*			*Aerococcus_Otu00053*
			*Enterococcus_77__Otu00071*			*Lactococcus_Otu00132*
			*Flavobacterium_Otu00354*			*Streptococcus_Otu00029*
			*Flavobacterium_Otu00138*			
			*Flavobacterium_Otu00160*			
			*Lactobacillus_Otu00092*			
			*Pseudomonas_Otu00173*			
			*Psychrobacter_Otu00047*			
V6	33	8	***Campylobacter_Otu00197***	24	4	***Escherichia_Shigella_Otu00046***
			***Campylobacter_Otu00233***			*Brochothrix_Otu00245*
			***Campylobacter_Otu00024***			*Carnobacterium_58__Otu00055*
			***Campylobacter_Otu00233***			*Pseudomonas_Otu00037*
			*Flavobacterium_Otu00160*			*Streptococcus_Otu00029*
			*Flavobacterium_Otu00354*			
			*Flavobacterium_Otu00138*			
			*Lactobacillus_Otu00092*			
			*Psychrobacter_Otu00047*			

Column: 1—Visit ID; 2—Total number of OTUs positively associated with one or more visits; 3—Number of OTUs positively associated with only one particular visit; 4—Potential spoilage or pathogen OTUs positively associated with a visit (potential pathogen bacteria are identified in bold); 5—Total number of OTUs negatively associated with one or more visits; 6—Number of OTUs negatively associated with only one particular visit; 7—Potential spoilage or pathogen OTUs negatively associated with one or more visits (potential pathogen bacteria are identified in bold). V1: Visit one; V2: Visit two; V3: Visit three; V4: Visit four; V5: Visit five; V6: Visit 6.

**Table 6:** Relevant bacteria (i.e., commonly known bacterial genera that can include potential spoilage species or potential foodborne pathogen species) extracted from the list of OTUs identified using the MaAsLin test and associated with the different production lines.

	Positive Associations			Negative Associations		
	Total	Unique	Relevant Bacterial Genera	Total	Unique	Relevant Bacterial Genera
CP	169	97	***Campylobacter_Otu00493***	13	2	*Acinetobacter_Otu00020*
			***Clostridium_sensu_stricto_Otu00016***			
			***Clostridium_sensu_stricto_Otu00136***			
			***Clostridium_sensu_stricto_Otu00307***			
			***Clostridium_sensu_stricto_Otu00441***			
			***Clostridium_sensu_stricto_Otu00059***			
			***Clostridium_XlVa_75__Otu00385***			
			*Enterococcus_77__Otu00071*			
			*Flavobacterium_Otu00480*			
			*Flavobacterium_Otu00333*			
			*Flavobacterium_Otu00160*			
			*Lactobacillus_Otu00092, Otu00099*			
			*Lactococcus_Otu00262, Otu00132*			
			*Moraxella_Otu00259*			
			*Pseudomonas_Otu00179, Otu00003*			
			*Psychrobacter_Otu00047*			
			*Psychrobacter_Otu00012*			
			*Psychrobacter_Otu00093*			
FL	42	14	***Clostridium_sensu_stricto_Otu00016***	42	2	*Acinetobacter_Otu00011, Otu0020*
						*Pseudomonas_Otu00003, Otu00037*
						*Psychrobacter_Otu00012, Otu00047*
						*Moraxella_Otu00045*
LO	36	11	***Clostridium_sensu_stricto_Otu00016***	40	4	*Acinetobacter_Otu00020, Otu00011*
			*Enterococcus_77__Otu00071*			*Moraxella_Otu00045*
			*Flavobacterium_Otu00138*			
			*Staphylococcus_99__Otu00008*			
BO	0	0		0	0	
PI	29	5	**Clostridium_sensu_stricto_97__Otu00203**	16	5	***Salmonella_66__Otu00026***
			*Psychrobacter_Otu00047*			*Acinetobacter_Otu00020, Otu00011*
						*Pseudomonas_Otu00023*
FE	45	8	***Clostridium_sensu_stricto_Otu00059***	21	0	*Moraxella_Otu00045*
			***Clostridium_sensu_stricto_Otu00016***			*Morganella*_91__Otu00337
			***Clostridium_sensu_stricto_Otu00136***			
			***Campylobacter_Otu00493***			
			***Escherichia_Shigella_Otu00046***			
			*Carnobacterium_58__Otu00055*			
			*Lactococcus_Otu00262*			
			*Psychrobacter_Otu00012*			

Column: 1—Production lines ID; 2—Total number of OTUs positively associated with one or more production lines; 3—Numbers of OTUs positively associated with only one particular production line; 4—Potential spoilage or pathogen OTUs positively associated with a production line (potential pathogen bacteria are identified in bold); 5—Total number of OTUs negatively associated with one or more production lines; 6—Number of OTUs negatively associated with only one particular production line; 7—Potential spoilage or pathogen OTUs negatively associated with a production line (potential pathogen bacteria are identified in bold); CP: Main production line; FL: Belly production line; LO: Loin production line; BO: Boston production line; PI: Picnic production line; FE: Ham production line.

## Data Availability

The data presented in this study are openly available in the NCBI Sequence Read Archive under accession number PRJNA758928 (https://www.ncbi.nlm.nih.gov/sra/PRJNA758928 (accessed on 1 September 2021).

## Supplementary Materials

The following supporting information can be downloaded at: https://www.mdpi.com/article/10.3390/microorganisms11010133/s1, Table S1: OTUs positively associated with visit according to the MaAsLin test; Table S2: OTUs negatively associated with visit according to the MaAsLin test; Table S3: OTUs positively associated with production line according to the MaAsLin test; Table S4: OTUs negatively associated with production line according to the MaAsLin test; Table S5: OTUs hierarchized according to their mean importance for the identification of a visit in the random forest visit model; Table S6: OTUs hierarchized according to their mean importance for the identification of a production line in the random forest production line model.
