# Supplementary material for: Distinct Microbiotas Are Associated with Different Production Lines in the Cutting Room of a Swine Slaughterhouse
Source: Microorganisms. 2023 Jan 4;11(1):133. doi: 10.3390/microorganisms11010133 (PMC9862343; doi:10.3390/microorganisms11010133)
Supplement: Supplementary file 1 [file microorganisms-11-00133-s001.zip › Supplementary_Figures_Text.pdf]

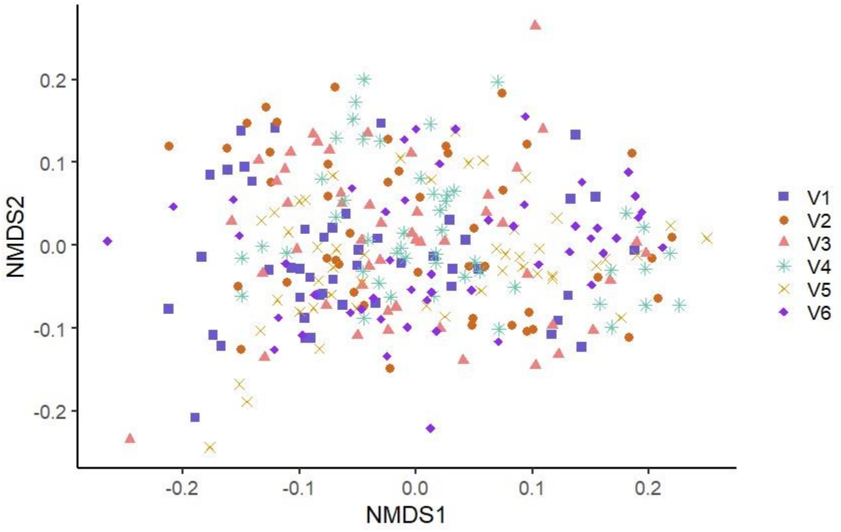

Figure S1. Non-Metric Multidimensional Scaling Graph (NMDS) of the microbiota structures of the six visit samples using the Jaccard index. Each point represents a sample, and each visit is represented by the combination of a symbol and a colour. Blue square: Visit one; Orange circle: Visit two; Pink triangle: Visit three; Turquoise star: Visit four; Yellow x: Visit five; Purple diamond: Visit six.

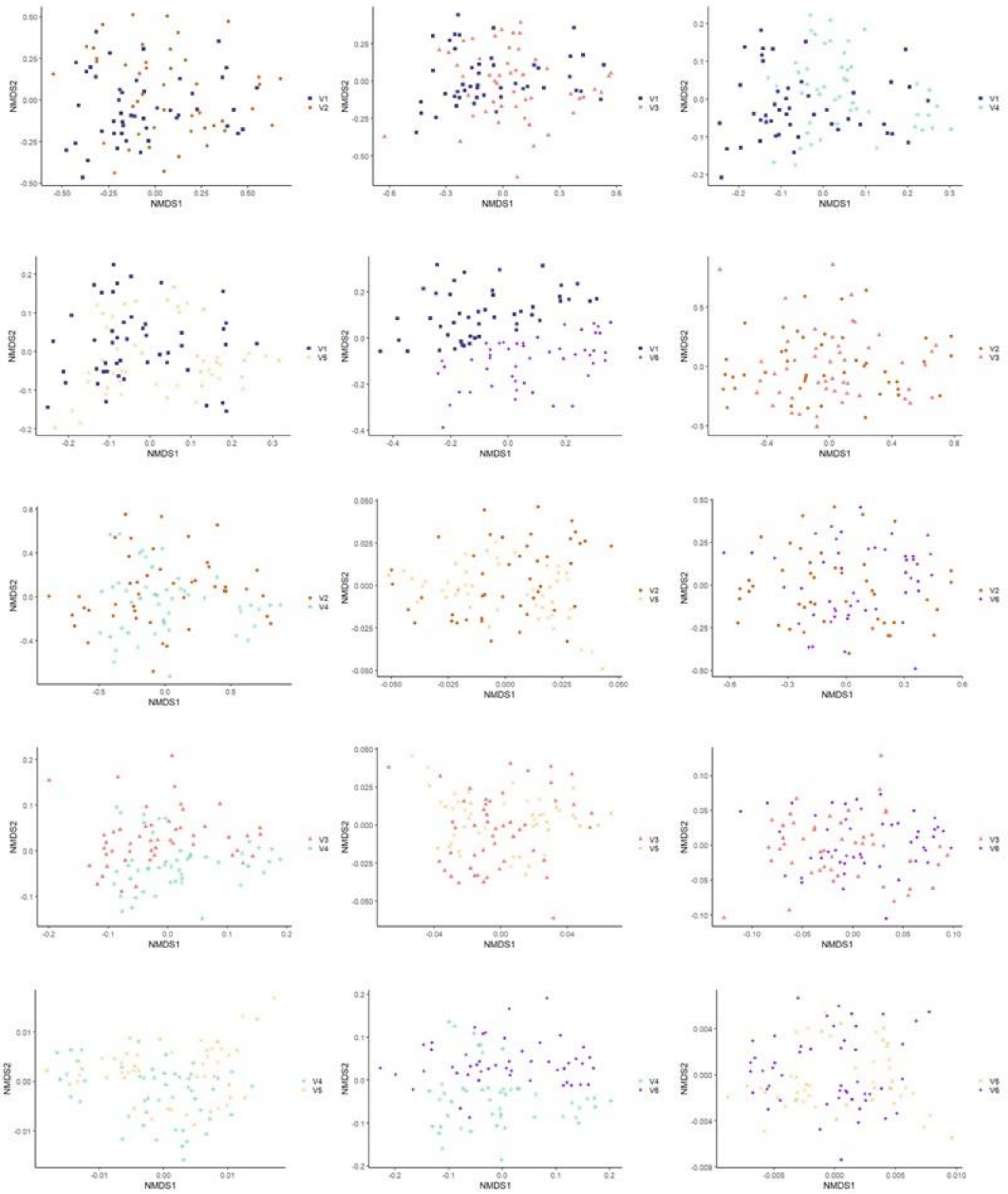

Figure S2. Non-Metric Multidimensional Scaling Graphs (NMDS) illustrating sample microbiome beta-diversity according to visit paired two by two using the Jaccard index. Blue square: Visit one; Orange circle: Visit two; Pink triangle: Visit three; Turquoise star: Visit four; Yellow x: Visit five; Purple diamond: Visit six.

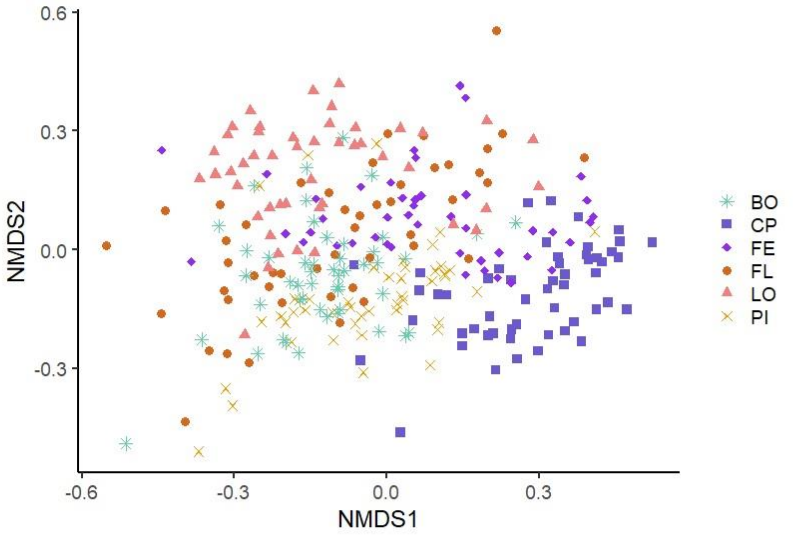

Figure S3. Non-Metric Multidimensional Scaling Graph (NMDS) of the microbiota structures of the six production line samples using Jaccard index. Each point represents a sample and each production line is represented by the combination of a symbol and a colour. Turquoise star: Boston production line (BO); Blue square: Main production line (CP); Purple diamond: Ham production line (FE); Orange circle: Belly production line (FL); Pink triangle: Loin production line (LO); Yellow x: Picnic production line (PI).

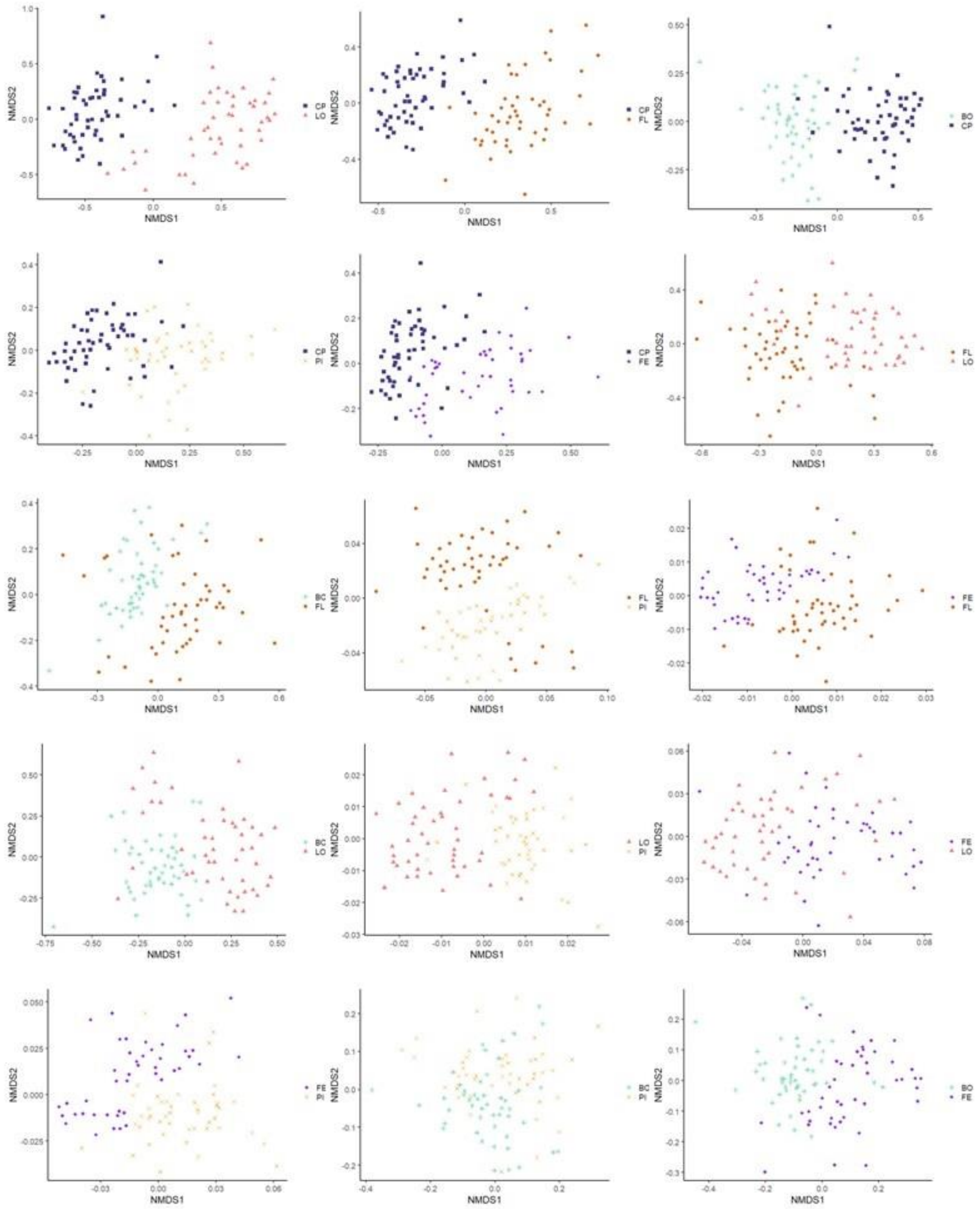

Figure S4. Non-Metric Multidimensional Scaling Graphs (NMDS) illustrating sample microbiome beta-diversity according to production line paired two by two using the Jaccard index. Turquoise star: Boston production line (BO); Blue square: Main production line (CP); Purple diamond: Ham production line (FE); Orange circle: Belly production line (FL); Pink triangle: Loin production line (LO); Yellow x: Picnic production line (PI).
