# Supplementary material for: Distinct Microbiotas Are Associated with Different Production Lines in the Cutting Room of a Swine Slaughterhouse
Source: Microorganisms. 2023 Jan 4;11(1):133. doi: 10.3390/microorganisms11010133 (PMC9862343; doi:10.3390/microorganisms11010133)
Supplement: Supplementary file 1 [file microorganisms-11-00133-s001.zip › Table S1. OTUs positively associated with visit according to the MaAsLin test.pdf]

Visit	Bacterial Genus and OTU
V2	*Actinomycetaceae_unclassified_99__Otu00164*
	Porphyromonas_Otu00295
	Comamonadaceae_unclassified_70__Otu00050
	Bacteria_unclassified_Otu00085
	Porphyromonadaceae_unclassified_84__Otu00220
	Porphyromonas_Otu00052
	Peptococcus_Otu00149
	Prevotella_Otu00283
	Porphyromonas_Otu00028
	Actinomycetales_unclassified_51__Otu00306
	Lactobacillales_unclassified_99__Otu00066
	Listeria_80__Otu00067
V3	*Porphyromonas_Otu00028*
	Porphyromonas_Otu00193
	Sandaracinobacter_Otu00158
	Clostridiales_Incertae_Sedis_XI_unclassified_Otu00221
	Flavobacteriaceae_unclassified_Otu00091
	Paracoccus_99__Otu00036
	Porphyromonas_Otu00052
	Brevundimonas_Otu00060
	Rhizobiaceae_unclassified_82__Otu00077
	Prevotella_Otu00495
	Psychrobacter_Otu00047
	Porphyromonas_Otu00013
	Roseomonas_99__Otu00328
	Acinetobacter_Otu00084
	Sphingobacterium_Otu00294
	Roseomonas_Otu00311
	Flavobacterium_Otu00354
	Peptococcus_Otu00149
	Rhodobacteraceae_unclassified_Otu00124
	Xanthobacteraceae_unclassified_67__Otu00174
	Clostridiales_unclassified_99__Otu00107
	Caulobacter_Otu00156
	Phenylobacterium_99__Otu00229
	Peptoniphilus_Otu00213
	Janthinobacterium_88__Otu00027
	Comamonadaceae_unclassified_70__Otu00050
	Fretibacterium_Otu00263
	Comamonadaceae_unclassified_95__Otu00114

	Devosia_Otu00405
	Clostridiales_unclassified_Otu00131
	Rhodobacteraceae_unclassified_97__Otu00133
	Amaricoccus_99__Otu00139
	Pseudomonas_Otu00173
	Azospira_Otu00476
	Parvimonas_Otu00014
	Mycoplasma_Otu00115
	Sphingobium_98__Otu00048
	Coriobacteriaceae_unclassified_Otu00110
	Rhizobiales_unclassified_98__Otu00276
V4	***Bergeyella_Otu00282***
	Chryseobacterium_Otu00025
	Psychrobacter_Otu00047
	Janthinobacterium_88__Otu00027
	Flavobacterium_Otu00138
	Aerococcaceae_unclassified_99__Otu00015
	Chryseobacterium_96__Otu00184
	Aquabacterium_59__Otu00222
	Polaromonas_60__Otu00078
	Acinetobacter_Otu00084
	Epilithonimonas_86__Otu00031
	Clostridiales_unclassified_99__Otu00107
	Lactobacillus_Otu00092
	Comamonadaceae_unclassified_70__Otu00050
	Ruminococcus2_80__Otu00338
	Neisseria_75__Otu00103
	Flavobacteriaceae_unclassified_Otu00091
	Fusobacterium_Otu00086
	Aerococcaceae_unclassified_Otu00010
	Peptoniphilus_Otu00213
	Clostridiales_unclassified_Otu00470
	Sphingobium_98__Otu00048
	Xanthomonadaceae_unclassified_Otu00195
	Actinobacillus_52__Otu00187
	Streptococcus_Otu00024
	Altererythrobacter_79__Otu00051
	Moraxellaceae_unclassified_Otu00349
	Xanthomonadaceae_unclassified_65__Otu00302
	Dietzia_Otu00398
	Amaricoccus_99__Otu00139

	Clostridiales_Incertae_Sedis_XI_unclassified_Otu00221
	Rhizobiaceae_unclassified_82__Otu00077
	Comamonadaceae_unclassified_95__Otu00114
	Lysobacter_99__Otu00057
	Peptococcus_Otu00149
	Enterococcus_77__Otu00071
	Caulobacter_Otu00156
	Azospira_Otu00476
	Brevundimonas_Otu00101
V5	***Corynebacterium_Otu00344***
	Porphyromonas_Otu00028
	Actinomycetaceae_unclassified_99__Otu00164
	Photobacterium_Otu00162
	Clostridiales_unclassified_Otu00336
	Aerococcaceae_unclassified_99__Otu00015
	Peptococcus_Otu00149
	Clostridiales_unclassified_99__Otu00107
	Flavobacterium_Otu00160
	Xanthobacteraceae_unclassified_67__Otu00174
	Janthinobacterium_88__Otu00027
	Campylobacter_Otu00197
	Rhodobacteraceae_unclassified_97__Otu00133
	Chitinophagaceae_unclassified_Otu00244
	Lactobacillales_unclassified_99__Otu00066
	Epilithonimonas_86__Otu00031
	Mycoplasma_Otu00115
	Pseudomonas_Otu00173
	Flavobacteriaceae_unclassified_Otu00091
	Anaerococcus_Otu00383
	Aerococcaceae_unclassified_Otu00010
	Porphyromonas_Otu00052
	Lactobacillus_Otu00092
	Psychrobacter_Otu00047
	Enterococcus_77__Otu00071
	Amaricoccus_99__Otu00139
	Brachybacterium_99__Otu00151
	Flavobacterium_Otu00354
	Eubacterium_Otu00097
	Prevotella_Otu00283
	Kurthia_99__Otu00120
	Duganella_96__Otu00106

	Clostridiales_Incertae_Sedis_XI_unclassified_Otu00221
	Paracoccus_99__Otu00036
	Altererythrobacter_79__Otu00051
	Comamonadaceae_unclassified_95__Otu00114
	Azospira_Otu00476
	Caulobacter_Otu00156
	Ruminococcus2_80__Otu00338
	Roseomonas_99__Otu00328
	Clostridiales_unclassified_Otu00131
	Chryseobacterium_Otu00025
	Roseomonas_Otu00311
	Sphingomonadaceae_unclassified_91__Otu00239
	Rhizobiales_unclassified_98__Otu00276
	Bosea_Otu00219
	Brochothrix_Otu00245
	Sphingomonas_Otu00043
	Flavobacterium_Otu00138
V6	*Parvimonas_Otu00014*
	Flavobacterium_Otu00160
	Bacteria_unclassified_Otu00404
	Psychrobacter_Otu00047
	Aerococcaceae_unclassified_99__Otu00015
	Rhodobacteraceae_unclassified_97__Otu00133
	Lactobacillales_unclassified_99__Otu00066
	Amaricoccus_99__Otu00139
	Flavobacterium_Otu00354
	Lactobacillus_Otu00092
	Prevotella_Otu00049
	Actinobacillus_52__Otu00187
	Janthinobacterium_88__Otu00027
	Peptoniphilus_Otu00005
	Campylobacter_Otu00197
	Sphaerobacteraceae_unclassified_Otu00279
	Campylobacter_Otu00233
	Epilithonimonas_86__Otu00031
	Streptococcus_Otu00024
	Ruminococcus2_80__Otu00338
	Duganella_96__Otu00106
	Mycoplasma_Otu00115
	Peptoniphilus_Otu00213
	Flavobacterium_Otu00138

Veillonellaceae_unclassified_Otu00488
Peptostreptococcus_Otu00018
Bacteria_unclassified_Otu00085
Porphyromonas_Otu00028
Paracoccus_99__Otu00036
Fretibacterium_Otu00263
Lysobacter_99__Otu00057
Prevotella_Otu00283
Fusobacterium_Otu00035

Table S1: OTUs positively associated with visit according to the MaAsLin test. Bacterial genera and OTUs associated with only one visit are identified in bold. 1: Visit one; 2: Visit two; 3: Visit three; 4: Visit four; 5: Visit five; 6: Visit six.
