# Supplementary material for: Distinct Microbiotas Are Associated with Different Production Lines in the Cutting Room of a Swine Slaughterhouse
Source: Microorganisms. 2023 Jan 4;11(1):133. doi: 10.3390/microorganisms11010133 (PMC9862343; doi:10.3390/microorganisms11010133)
Supplement: Supplementary file 1 [file microorganisms-11-00133-s001.zip › Table S2 OTUs negatively associated with visit according to the MaAsLin test.pdf]

Visit	Bacterial Genus and OTU
V2	*Prevotella_Otu00117*
	Peptostreptococcaceae_unclassified_99__Otu00266
	Bacteria_unclassified_Otu00297
	Lachnospiraceae_unclassified_Otu00069
	Helcococcus_Otu00080
	Filifactor_Otu00230
	Bacteroides_Otu00009
	Helcococcus_Otu00104
	Fusobacterium_Otu00001
	Pasteurella_83__Otu00121
	Streptococcus_Otu00029
	Parvimonas_Otu00014
	Tannerella_Otu00210
	Fusobacterium_Otu00396
	Porphyromonadaceae_unclassified_99__Otu00089
	Brochothrix_Otu00245
	Clostridiales_unclassified_96__Otu00275
	Escherichia_Shigella_Otu00046
	Kurthia_99__Otu00120
	Bacillales_unclassified_91__Otu00061
V3	*Prevotella_Otu00117*
	Lachnospiraceae_unclassified_Otu00069
	Peptostreptococcaceae_unclassified_99__Otu00266
	Porphyromonadaceae_unclassified_99__Otu00089
	Bacilli_unclassified_Otu00224
	Bacteria_unclassified_Otu00297
	Streptococcus_Otu00029
	Helcococcus_Otu00080
	Bacteroides_Otu00009
	Lactococcus_Otu00132
	Filifactor_Otu00230
	Peptostreptococcus_Otu00018
	Fusobacterium_Otu00396
	Lactobacillales_unclassified_99__Otu00066
	Bacillales_unclassified_91__Otu00061
	Prevotella_Otu00439
	Fusobacterium_Otu00035
	Blastococcus_91__Otu00247
	Helcococcus_Otu00181
	Enhydrobacter_Otu00007

V4	*Prevotella_Otu00117*
	Lachnospiraceae_unclassified_Otu00069
	Bacteria_unclassified_Otu00297
	Bacteroides_Otu00009
	Peptostreptococcaceae_unclassified_99__Otu00266
	Bacilli_unclassified_Otu00224
	Fusobacterium_Otu00001
	Actinomyces_Otu00068
	Escherichia_Shigella_Otu00046
	Filifactor_Otu00230
	Bacteroides_Otu00030
	Lachnospiraceae_unclassified_Otu00095
	BPrevotella_Otu00049
	Helcococcus_Otu00104
	Clostridiales_Incertae_Sedis_XI_unclassified_Otu00218
	Atopobium_Otu00090
	Coriobacteriaceae_unclassified_Otu00110
	Aerococcus_Otu00053
	Bacillales_unclassified_91__Otu00061
	Pasteurella_83__Otu00121
	Filifactor_Otu00137
	Lactococcus_Otu00132
	Helcococcus_Otu00080
	Peptostreptococcus_Otu00018
	Bacteroides_Otu00017
	Fusobacterium_Otu00035
	Tannerella_Otu00210
	Porphyromonadaceae_unclassified_99__Otu00089
V5	*Prevotella_Otu00117*
	Peptostreptococcaceae_unclassified_99__Otu00266
	Fusobacterium_Otu00396
	Filifactor_Otu00230
	Bacilli_unclassified_Otu00224
	Streptococcus_Otu00029
	Lachnospiraceae_unclassified_Otu00069
	Aeromonas_Otu00062
	Bacteroides_Otu00009
	Peptostreptococcus_Otu00018
	Clostridiales_Incertae_Sedis_XI_unclassified_Otu00218
	Escherichia_Shigella_Otu00046
	Bacillales_unclassified_91__Otu00061

	Helcococcus_Otu00181
	Blastococcus_91__Otu00247
	Aerococcus_Otu00053
	Lactococcus_Otu00132
	Chryseobacterium_Otu00064
	Fusobacterium_Otu00001
	Porphyromonadaceae_unclassified_99__Otu00089
	Actinomyces_Otu00068
	Helcococcus_Otu00104
V6	*Prevotella_Otu00117*
	Peptostreptococcaceae_unclassified_99__Otu00266
	Bacteria_unclassified_Otu00297
	Porphyromonadaceae_unclassified_99__Otu00089
	Lachnospiraceae_unclassified_Otu00069
	Escherichia_Shigella_Otu00046
	Bacteroides_Otu00017
	Carnobacterium_58__Otu00055
	Tannerella_Otu00210
	Fusobacterium_Otu00396
	Pseudomonas_Otu00037
	Aeromonas_Otu00062
	Kurthia_99__Otu00120
	Enhydrobacter_Otu00007
	Bacteroides_Otu00009
	Streptococcus_Otu00029
	Brevundimonas_Otu00072
	Brevundimonas_Otu00101
	Brochothrix_Otu00245
	Lachnospiraceae_unclassified_Otu00095
	Fusobacterium_Otu00001
	Bacteroides_Otu00030
	Atopobium_Otu00090
	Clostridiales_unclassified_96__Otu00275

Table S2: OTUs negatively associated with visit according to the MaAsLin test. Bacterial genera and OTUs associated with only one visit are identified in bold. 1: Visit one; 2: Visit two; 3: Visit three; 4: Visit four; 5: Visit five; 6: Visit six.
