# Supplementary material for: Distinct Microbiotas Are Associated with Different Production Lines in the Cutting Room of a Swine Slaughterhouse
Source: Microorganisms. 2023 Jan 4;11(1):133. doi: 10.3390/microorganisms11010133 (PMC9862343; doi:10.3390/microorganisms11010133)
Supplement: Supplementary file 1 [file microorganisms-11-00133-s001.zip › Table S3 OTUs positively associated with production line according to the MaAsLin test.pdf]

Production line	Bacterial Genus and OTU
CP	*Rothia_92__Otu00006*
	Desulfovibrio_Otu00281
	Lactobacillales_unclassified_99__Otu00515
	Bacteroides_Otu00258
	Bacteroidetes_unclassified_Otu00453
	Dechloromonas_90__Otu00175
	Clostridiaceae_1_unclassified_Otu00082
	Streptococcus_Otu00232
	Rhodocyclaceae_unclassified_85__Otu00285
	Bacteroides_Otu00199
	Stenotrophomonas_99__Otu00054
	Leucobacter_98__Otu00409
	Mannheimia_93__Otu00458
	Paludibacter_Otu00166
	Flavobacteriaceae_unclassified_Otu00201
	Lactococcus_Otu00262
	Propionivibrio_96__Otu00373
	Gallicola_Otu00249
	Deinococcus_Otu00461
	Acidaminococcus_Otu00393
	Rhodopseudomonas_99__Otu00033
	Turicibacter_Otu00058
	Polaromonas_60__Otu00078
	Soonwooa_Otu00196
	Cloacibacterium_Otu00172
	Azonexus_Otu00322
	Lactobacillus_Otu00099
	Romboutsia_Otu00075
	Terrisporobacter_Otu00041
	Clostridium_sensu_stricto_Otu00016
	Microvirgula_Otu00206
	Pseudomonas_Otu00179
	Bacteroides_Otu00264
	Flavobacterium_Otu00480
	Clostridium_sensu_stricto_Otu00136
	Aerococcaceae_unclassified_Otu00010
	Bacteroidales_unclassified_88__Otu00482
	Neisseriaceae_unclassified_Otu00442
	Brevibacterium_Otu00142

Moraxellaceae_unclassified_Otu00314
Methanobrevibacter_Otu00183
Dysgonomonas_Otu00363
Dysgonomonas_Otu00484
Lachnospiraceae_unclassified_87__Otu00572
Psychrobacter_Otu00047
Ruminococcaceae_unclassified_Otu00154
Porphyromonas_98__Otu00454
Clostridium_sensu_stricto_Otu00307
Helcococcus_Otu00399
Prevotella_Otu00272
Flavobacterium_Otu00333
Coprococcus_Otu00246
Porphyromonadaceae_unclassified_63__Otu00417
Prevotella_Otu00240
Lactobacillus_Otu00092
Chryseobacterium_Otu00042
Campylobacter_Otu00493
Peptococcus_Otu00407
Vitreoscilla_93__Otu00391
Psychrobacter_Otu00012
Peptostreptococcus_Otu00186
Lachnospiraceae_unclassified_Otu00253
Prevotella_Otu00347
Corynebacterium_Otu00100
Nosocomiicoccus_92__Otu00291
Microbacterium_73__Otu00433
Lachnospiraceae_unclassified_Otu00446
Proteocatella_Otu00348
Treponema_Otu00449
Sanguibacter_93__Otu00191
Blautia_Otu00360
Empedobacter_Otu00352
Psychrobacter_Otu00093
Arcobacter_Otu00273
Peptostreptococcaceae_unclassified_98__Otu00452
Facklamia_92__Otu00130
Butyricicoccus_Otu00325
Clostridiales_unclassified_Otu00131
Prevotella_Otu00520
Phascolarctobacterium_Otu00211

Chryseobacterium_Otu00144
Bacteroidetes_unclassified_Bacteroidetes_unclassified_Otu00370
Clostridium_XlVa_75__Otu00385
Porphyromonadaceae_unclassified_Otu00289
Coprococcus_Otu00424
Moraxella_Otu00259
Facklamia_99__Otu00254
Comamonadaceae_unclassified_70__Otu00050
Kocuria_95__Otu00094
Porphyromonadaceae_unclassified_Otu00414
Lactococcus_Otu00132
Fastidiosipila_Otu00225
Firmicutes_unclassified_Otu00256
Ruminococcus_99__Otu00428
Clostridiales_unclassified_88__Otu00235
Porphyromonas_Otu00358
Bacteroides_Otu00241
Brevundimonas_Otu00101
Roseburia_96__Otu00280
Clostridiales_Incertae_Sedis_XI_unclassified_Otu00157
Lachnospiraceae_unclassified_Otu00364
Sphingopyxis_Otu00231
Ruminococcaceae_unclassified_85__Otu00422
Catenibacterium_Otu00317
Porphyromonadaceae_unclassified_Otu00234
Firmicutes_unclassified_99__Otu00546
Prevotella_Otu00159
Ruminococcaceae_unclassified_93__Otu00153
Collinsella_Otu00527
Blautia_Otu00361
Ruminococcaceae_unclassified_Otu00427
Ruminococcaceae_unclassified_97__Otu00270
Mycobacterium_Otu00116
Clostridiales_unclassified_84__Otu00321
Intestinimonas_Otu00402
Butyricicoccus_Otu00312
Streptococcus_Otu00237
Methanobrevibacter_Otu00413
Holdemanella_Otu00594
Blautia_Otu00392
Anaerococcus_Otu00202

Ruminococcaceae_unclassified_Otu00287
Prevotella_Otu00471
Ruminococcaceae_unclassified_95__Otu00451
Microbacteriaceae_unclassified_91__Otu00135
Porphyromonas_Otu00223
Clostridium_sensu_stricto_Otu00441
Rhizobium_Otu00345
Blautia_98__Otu00381
Collinsella_Otu00353
Clostridiales_unclassified_Otu00326
Peptoniphilus_Otu00213
Flavobacterium_Otu00160
Methanosphaera_Otu00379
Clostridiales_unclassified_99__Otu00107
Ruminococcaceae_unclassified_Otu00320
Bulleidia_Otu00450
Actinomycetales_unclassified_66__Otu00464
Chryseobacterium_Otu00064
Azospira_Otu00476
Exiguobacterium_99__Otu00127
Ruminococcaceae_unclassified_Otu00456
Clostridium_sensu_stricto_Otu00059
Lachnospiraceae_unclassified_Otu00069
Ezakiella_Otu00150
Lachnospiraceae_unclassified_Otu00355
Peptoniphilus_Otu00105
Peptostreptococcaceae_unclassified_65__Otu00387
Mitsuokella_96__Otu00309
Neisseria_75__Otu00103
Propionispira_87__Otu00217
Bifidobacterium_Otu00299
Rhodococcus_Otu00126
Firmicutes_unclassified_Otu00362
Bacteroidales_unclassified_Otu00257
Comamonas_54__Otu00087
Enhydrobacter_Otu00007
Exiguobacterium_Otu00334
Ruminococcus_69__Otu00330
Enterococcus_77__Otu00071
Prevotella_Otu00269
Peptococcus_Otu00149

	Pseudomonas_Otu00003
	Streptococcus_Otu00024
	Flavobacteriaceae_unclassified_Otu00316
	Pedobacter_Otu00265
	Clostridiales_Incertae_Sedis_XI_unclassified_Otu00221
	Ruminococcaceae_unclassified_Otu00209
	Anaerovibrio_81__Otu00489
FL	***Clostridiales_unclassified_74__Otu00215***
	Anaerococcus_Otu00076
	Corynebacterium_Otu00242
	Ezakiella_Otu00208
	Peptoniphilus_Otu00141
	Peptoniphilus_Otu00105
	Porphyromonas_Otu00152
	Clostridiales_Incertae_Sedis_XI_unclassified_Otu00250
	Anaerococcus_Otu00216
	Clostridiales_Incertae_Sedis_XI_unclassified_Otu00157
	Ezakiella_Otu00150
	Peptoniphilaceae_unclassified_Otu00140
	Clostridiales_Incertae_Sedis_XI_Finegoldia_Otu00163
	Mobiluncus_Otu00323
	Micrococcaceae_unclassified_98__Otu00207
	Clostridiales_unclassified_99__Otu00107
	Corynebacterium_Otu00081
	Fastidiosipila_Otu00225
	Aerococcaceae_unclassified_Otu00010
	Duganella_96__Otu00106
	Peptococcus_Otu00149
	Peptostreptococcaceae_unclassified_98__Otu00452
	Anaerococcus_Otu00202
	Atopostipes_Otu00176
	Ruminococcaceae_unclassified_Otu00154
	Anaerococcus_Otu00383
	Phenylobacterium_Otu00056
	Facklamia_92__Otu00130
	Gemmata_Otu00236
	Facklamia_99__Otu00254
	Porphyromonas_Otu00358
	Collinsella_Otu00527
	Nosocomiicoccus_92__Otu00291
	Clostridiales_unclassified_Otu00131

	Xanthomonadaceae_unclassified_65__Otu00302
	Helcococcus_Otu00399
	Macrococcus_99__Otu00019
	Clostridium_sensu_stricto_Otu00016
	Trueperella_Otu00002
	Clostridiales_Incertae_Sedis_XI_unclassified_Otu00221
	Methanosphaera_Otu00379
	Peptoniphilus_Otu00213
LO	***Kurthia_99__Otu00120***
	Enterococcus_77__Otu00071
	Phenylobacterium_Otu00056
	Bradyrhizobium_Otu00044
	Staphylococcus_99__Otu00008
	Rothia_Otu00346
	Duganella_96__Otu00106
	Macrococcus_99__Otu00019
	Aerococcaceae_unclassified_Otu00010
	Corynebacterium_Otu00081
	Blactococcus_Otu00132
	Gemmata_Otu00236
	Peptostreptococcus_Otu00186
	Clostridiaceae_1_unclassified_Otu00082
	Pelomonas_65__Otu00128
	Flavobacterium_Otu00138
	Enhydrobacter_Otu00007
	Clostridium_sensu_stricto_Otu00016
	Ruminococcaceae_unclassified_Otu00154
	Rothia_92__Otu00006
	Nocardioides_Otu00342
	Micrococcaceae_unclassified_Otu00038
	Neisseria_75__Otu00103
	Ruminococcaceae_unclassified_93__Otu00153
	Clostridiales_unclassified_Otu00131
	Porphyromonadaceae_unclassified_84__Otu00220
	Mobiluncus_Otu00323
	Corynebacterium_Otu00242
	Chryseobacterium_Otu00064
	Romboutsia_Otu00075
	Brachybacterium_99__Otu00151
	Comamonas_54__Otu00087
	Peptoniphilus_Otu00105

	Janthinobacterium_88__Otu00027
	Bacteroides_Otu00241
	Veillonella_Otu00111
PI	*Psychrobacter_Otu00047*
	Atopobium_Otu00090
	Rothia_92__Otu00006
	Clostridiaceae_1_unclassified_Otu00082
	Aerococcaceae_unclassified_Otu00010
	Rhodopseudomonas_99__Otu00033
	Clostridiales_Incertae_Sedis_XI_unclassified_Otu00157
	Ruminococcaceae_unclassified_Otu00154
	Flavobacteriaceae_Soonwooa_Otu00196
	Lachnospiraceae_unclassified_Otu00069
	Facklamia_92__Otu00130
	Porphyromonas_98__Otu00454
	Peptoniphilus_Otu00105
	Chryseobacterium_Otu00144
	Gallicola_Otu00249
	Empedobacter_Otu00352
	Ruminococcaceae_unclassified_Otu00209
	Clostridiales_unclassified_99__Otu00107
	Helcococcus_Otu00399
	Fastidiosipila_Otu00225
	Clostridiales_unclassified_Otu00131
	Peptostreptococcus_Otu00018
	Vitreoscilla_93__Otu00391
	Clostridiales_unclassified_88__Otu00235
	Ezakiella_Otu00150
	Microvirgula_Otu00206
	Facklamia_99__Otu00254
	Bacteroides_Otu00022
	Clostridium_sensu_stricto_97__Otu00203
FE	*Psychrobacter_Otu00012*
	Morganella_91__Otu00337
	Escherichia_Shigella_Otu00046
	Carnobacterium_58__Otu00055
	Catenibacterium_Otu00317
	Arthrobacter_Otu00063
	Macrococcus_99__Otu00019
	Exiguobacterium_99__Otu00127
	Prevotella_Otu00240
	Clostridium_sensu_stricto_Otu00059

Janthinobacterium_88__Otu00027
Phascolarctobacterium_Otu00211
Firmicutes_unclassified_Otu00256
Rothia_92__Otu00006
Ruminococcaceae_unclassified_94__Otu00351
Prevotella_Otu00347
Clostridium_sensu_stricto_Otu00016
Coprococcus_Otu00246
Ruminococcus_69__Otu00330
Ruminococcaceae_unclassified_93__Otu00153
Bifidobacterium_Otu00299
Roseburia_96__Otu00280
Anaerovibrio_81__Otu00489
Terrisporobacter_Otu00041
Blautia_98__Otu00381
Methanobrevibacter_Otu00183
Lactococcus_Otu00262
Lachnospiraceae_unclassified_Otu00446
Campylobacter_Otu00493
Ruminococcaceae_unclassified_Otu00427
Clostridiaceae_1_unclassified_Otu00082
Porphyromonadaceae_unclassified_Otu00289
Clostridiales_unclassified_84__Otu00321
Duganella_96__Otu00106
Prevotella_Otu00159
Vagococcus_93__Otu00278
Porphyromonadaceae_unclassified_Otu00234
Clostridium_sensu_stricto_Otu00136
BCoprococcus_Otu00424
Ruminococcaceae_unclassified_97__Otu00270
Mitsuokella_96__Otu00309
Blautia_Otu00361
Porphyromonadaceae_unclassified_84__Otu00220
Clostridium_XlVa_75__Otu00385
Aeromonas_Otu00062

Table S3: OTUs positively associated with production line according to the MaAsLin test. Bacterial genera and OTUs associated with only one production line are identified in bold. CP: Main production line; FL: Belly production line; LO: Loin production line; BO: Boston production line; PI: Picnic production line; FE: Ham production line.
