# Supplementary material for: Distinct Microbiotas Are Associated with Different Production Lines in the Cutting Room of a Swine Slaughterhouse
Source: Microorganisms. 2023 Jan 4;11(1):133. doi: 10.3390/microorganisms11010133 (PMC9862343; doi:10.3390/microorganisms11010133)
Supplement: Supplementary file 1 [file microorganisms-11-00133-s001.zip › Table S4 OTUs negatively associated with production line according to the MaAsLin test.pdf]

Production line	Bacterial Genus and OTU
CP	*Fusobacterium_Otu00001*
	Mycoplasma_Otu00112
	Bacteria_unclassified_Otu00085
	Bacteroides_Otu00022
	Porphyromonas_Otu00295
	Helcococcus_Otu00080
	Trueperella_Otu00002
	Porphyromonas_Otu00079
	Eubacterium_Otu00097
	Peptoniphilus_Otu00005
	Acinetobacter_Otu00020
	Pseudomonas_Otu00037
	Micrococcaceae_unclassified_Otu00038
FL	*Pasteurellaceae_unclassified_Otu00034*
	Moraxella_Otu00045
	Moraxellaceae_unclassified_91__Otu00122
	Neisseriaceae_unclassified_99__Otu00065
	Porphyromonas_58__Otu00109
	Mycoplasma_Otu00112
	Porphyromonas_Otu00295
	Porphyromonas_Otu00079
	Bacteroidales_unclassified_96__Otu00161
	Bacteroidales_unclassified_Otu00188
	Corynebacterium_99__Otu00251
	Bacteroides_Otu00022
	Acinetobacter_Otu00011
	Chryseobacterium_Otu00042
	Pseudomonas_Otu00003
	Leptotrichiaceae_unclassified_Otu00286
	Psychrobacter_Otu00012
	Gemella_Otu00134
	Porphyromonas_Otu00052
	Flavobacteriaceae_unclassified_99__Otu00155
	Porphyromonadaceae_unclassified_99__Otu00089
	Bacteria_unclassified_Otu00260
	Flavobacteriaceae_unclassified_Otu00316
	Lactobacillales_unclassified_98__Otu00356
	Chryseobacterium_Otu00025
	Eubacterium_Otu00097

	Rhodopseudomonas_99__Otu00033
	Prevotellaceae_unclassified_Otu00248
	Clostridiales_unclassified_Otu00336
	Lachnospiraceae_unclassified_Otu00146
	Fusobacterium_Otu00001
	Pseudomonas_Otu00037
	Psychrobacter_Otu00047
	Aeromonas_Otu00062
	Porphyromonas_Otu00032
	Helcococcus_Otu00080
	Porphyromonas_Otu00013
	Acinetobacter_Otu00020
	Bacteria_unclassified_Otu00118
	Fretibacterium_Otu00263
	Coriobacteriaceae_unclassified_Otu00110
	Fusobacterium_Otu00086
LO	*Bacteroides_Otu00022*
	Moraxella_Otu00045
	Moraxellaceae_unclassified_91__Otu00122
	Neisseriaceae_unclassified_99__Otu00065
	Fusobacterium_Otu00001
	Pasteurellaceae_unclassified_Otu00034
	Mycoplasma_Otu00112
	Helcococcus_Otu00080
	Acinetobacter_Otu00020
	Trueperella_Otu00002
	Porphyromonas_Otu00295
	Porphyromonas_58__Otu00109
	Corynebacterium_99__Otu00251
	Porphyromonas_Otu00079
	Eubacterium_Otu00097
	Fusobacterium_Otu00102
	Porphyromonas_Otu00032
	Bacteroides_Otu00009
	Bacteroidales_unclassified_Otu00188
	Peptostreptococcus_Otu00018
	Coriobacteriaceae_unclassified_Otu00110
	Porphyromonadaceae_unclassified_99__Otu00089
	Porphyromonas_Otu00013
	Peptoniphilus_Otu00005
	Bacteria_unclassified_Otu00260

	Acinetobacter_Otu00011
	Bacteroidales_unclassified_96__Otu00161
	Flavobacteriaceae_unclassified_99__Otu00155
	Prevotellaceae_unclassified_Otu00248
	Leptotrichiaceae_unclassified_Otu00286
	Chryseobacterium_Otu00042
	Lactobacillales_unclassified_98__Otu00356
	Lachnospiraceae_unclassified_Otu00146
	Flavobacteriaceae_unclassified_Otu00316
	Pseudoxanthomonas_Otu00339
	Gemella_Otu00134
	Rhodopseudomonas_99__Otu00033
	Streptococcus_Otu00237
	Filifactor_Otu00137
	Fretibacterium_Otu00263
PI	*Acinetobacter_Otu00020*
	Chryseobacterium_Otu00025
	Sphingomonas_Otu00070
	Pseudomonas_Otu00023
	Acinetobacter_Otu00011
	Actinomycetales_unclassified_51__Otu00306
	Salmonella_66__Otu00026
	Aeromonas_Otu00062
	Porphyromonas_Otu00295
	Pseudoxanthomonas_Otu00339
	Moraxellaceae_unclassified_91__Otu00122
	Gemella_Otu00134
	Fusobacterium_Otu00001
	Bacteria_unclassified_Otu00118
	Janthinobacterium_88__Otu00027
	Fretibacterium_Otu00263
FE	*Bacteroides_Otu00022*
	Pasteurellaceae_unclassified_Otu00034
	Helcococcus_Otu00080
	Fusobacterium_Otu00001
	Moraxellaceae_unclassified_91__Otu00122
	Mycoplasma_Otu00112
	Trueperella_Otu00002
	Eubacterium_Otu00097
	Chryseobacterium_Otu00025
	Bacteroides_Otu00009

Porphyromonas_Otu00295
Moraxella_Otu00045
Corynebacterium_99__Otu00251
Bacteria_unclassified_Otu00118
Porphyromonas_Otu00013
Neisseriaceae_unclassified_99__Otu00065
Coriobacteriaceae_unclassified_Otu00110
Gemella_Otu00134
Clostridiales_unclassified_Otu00336
Porphyromonas_58__Otu00109
Bacteroidales_unclassified_Otu00188

Table S4: OTUs negatively associated with production line according to the MaAsLin test. Bacterial genera and OTUs associated with only one production line are identified in bold. CP: Main production line; FL: Belly production line; LO: Loin production line; BO: Boston production line; PI: Picnic production line; FE: Ham production line.
