# Supplementary material for: Distinct Microbiotas Are Associated with Different Production Lines in the Cutting Room of a Swine Slaughterhouse
Source: Microorganisms. 2023 Jan 4;11(1):133. doi: 10.3390/microorganisms11010133 (PMC9862343; doi:10.3390/microorganisms11010133)
Supplement: Supplementary file 1 [file microorganisms-11-00133-s001.zip › Table S5 OTUs hierarchized according to their mean importance for the identification of a visit in the random forest visit model.pdf]

Table S5: OTUs hierarchized according to their mean importance for the identification of a visit in the random forest visit model.

Rank	OTU	Bacterial Genus	Importance
1	Otu00117	*Prevotella*	0.00754902
2	Otu00015	*Aerococcaceae_unclassified*	0.00665979
3	Otu00014	*Parvimonas*	0.00644643
4	Otu00344	*Corynebacterium*	0.00593278
5	Otu00049	*Prevotella*	0.00557527
6	Otu00066	*Lactobacillales_unclassified*	0.00547569
7	Otu00028	*Porphyromonas*	0.00437562
8	Otu00017	*Bacteroides*	0.00436419
9	Otu00069	*Lachnospiraceae_unclassified*	0.00432695
10	Otu00164	*Actinomycetaceae_unclassified*	0.00389317
11	Otu00224	*Bacilli_unclassified*	0.00382197
12	Otu00297	*Bacteria_unclassified*	0.00373274
13	Otu00266	*Peptostreptococcaceae_unclassified*	0.00343795
14	Otu00035	*Fusobacterium*	0.00318827
15	Otu00001	*Fusobacterium*	0.00313769
16	Otu00018	*Peptostreptococcus*	0.00297853
17	Otu00046	*Escherichia/Shigella*	0.002933
18	Otu00162	*Photobacterium*	0.00292434
19	Otu00080	*Helcococcus*	0.00286206
20	Otu00115	*Mycoplasma*	0.00277772
21	Otu00160	*Flavobacterium*	0.00267869
22	Otu00029	*Streptococcus*	0.00266186
23	Otu00025	*Chryseobacterium*	0.00266052
24	Otu00005	*Peptoniphilus*	0.00265971
25	Otu00031	*Epilithonimonas*	0.00264622
26	Otu00052	*Porphyromonas*	0.0025355
27	Otu00193	*Porphyromonas*	0.00242993
28	Otu00009	*Bacteroides*	0.00242468
29	Otu00181	*Helcococcus*	0.00220673
30	Otu00230	*Filifactor*	0.00214084
31	Otu00047	*Psychrobacter*	0.00211189
32	Otu00097	*Eubacterium*	0.00209746
33	Otu00030	*Bacteroides*	0.00202445
34	Otu00197	*Campylobacter*	0.00200184
35	Otu00404	*Bacteria_unclassified*	0.0019922
36	Otu00022	*Bacteroides*	0.00196726
37	Otu00336	*Clostridiales_unclassified*	0.00193741
38	Otu00032	*Porphyromonas*	0.00185845
39	Otu00120	*Kurthia*	0.00182929

40	Otu00013	*Porphyromonas*	0.00180634
41	Otu00062	*Aeromonas*	0.00178152
42	Otu00104	*Helcococcus*	0.00175999
43	Otu00027	*Janthinobacterium*	0.00174556
44	Otu00085	*Bacteria_unclassified*	0.00174097
45	Otu00089	*Porphyromonadaceae_unclassified*	0.00163324
46	Otu00055	*Carnobacterium*	0.00162044
47	Otu00282	*Bergeyella*	0.00157329
48	Otu00078	*Polaromonas*	0.00156524
49	Otu00121	*Pasteurella*	0.00156145
50	Otu00090	*Atopobium*	0.0015574
51	Otu00368	*Schlegelella*	0.00155714
52	Otu00218	*Clostridiales_Incertae_Sedis_XI_unclassified*	0.00155259
53	Otu00138	*Flavobacterium*	0.00153083
54	Otu00071	*Enterococcus*	0.00148827
55	Otu00020	*Acinetobacter*	0.00145952
56	Otu00002	*Trueperella*	0.00136781
57	Otu00019	*Macrococcus*	0.00134792
58	Otu00024	*Streptococcus*	0.00134049
59	Otu00210	*Tannerella*	0.00132554
60	Otu00092	*Lactobacillus*	0.00132091
61	Otu00235	*Clostridiales_unclassified*	0.00131959
62	Otu00103	*Neisseria*	0.00130282
63	Otu00174	*Xanthobacteraceae_unclassified*	0.00128876
64	Otu00110	*Coriobacteriaceae_unclassified*	0.00124826
65	Otu00037	*Pseudomonas*	0.00123504
66	Otu00011	*Acinetobacter*	0.00123326
67	Otu00016	*Clostridium_sensu_stricto*	0.00122564
68	Otu00154	*Ruminococcaceae_unclassified*	0.00122006
69	Otu00084	*Acinetobacter*	0.00113304
70	Otu00101	*Brevundimonas*	0.0011283
71	Otu00010	*Aerococcaceae_unclassified*	0.00111641
72	Otu00133	*Rhodobacteraceae_unclassified*	0.00109471
73	Otu00099	*Lactobacillus*	0.00107702
74	Otu00056	*Phenylobacterium*	0.00107302
75	Otu00006	*Rothia*	0.00106707
76	Otu00068	*Actinomyces*	0.00105174
77	Otu00050	*Comamonadaceae_unclassified*	0.00103582
78	Otu00122	*Moraxellaceae_unclassified*	0.00103328
79	Otu00060	*Brevundimonas*	0.00101517
80	Otu00077	*Rhizobiaceae_unclassified*	0.001013
81	Otu00007	*Enhydrobacter*	0.00101295

82	Otu00012	*Psychrobacter*	0.00098394
83	Otu00095	*Lachnospiraceae_unclassified*	0.00097684
84	Otu00149	*Peptococcus*	0.00094882
85	Otu00158	*Sandaracinobacter*	0.00093348
86	Otu00132	*Lactococcus*	0.00091831
87	Otu00107	*Clostridiales_unclassified*	0.00090597
88	Otu00221	*Clostridiales_Incertae_Sedis_XI_unclassified*	0.00088122
89	Otu00082	*Clostridiaceae_1_unclassified*	0.00086872
90	Otu00064	*Chryseobacterium*	0.0008601
91	Otu00517	*Micrococcaceae_unclassified*	0.00085327
92	Otu00332	*Odoribacter*	0.00084379
93	Otu00245	*Brochothrix*	0.00084185
94	Otu00112	*Mycoplasma*	0.00083828
95	Otu00377	*Catonella*	0.00083762
96	Otu00139	*Amaricoccus*	0.00083541
97	Otu00008	*Staphylococcus*	0.00081037
98	Otu00102	*Fusobacterium*	0.00079038
99	Otu00354	*Flavobacterium*	0.00078212
100	Otu00651	*Lachnoanaerobaculum*	0.00076333
101	Otu00036	*Paracoccus*	0.00076203
102	Otu00091	*Flavobacteriaceae_unclassified*	0.00075036
103	Otu00042	*Chryseobacterium*	0.00073935
104	Otu00079	*Porphyromonas*	0.00073418
105	Otu01118	*Bacteroides*	0.00071537
106	Otu00137	*Filifactor*	0.00071361
107	Otu00240	*Prevotella*	0.00071091
108	Otu00130	*Facklamia*	0.00070741
109	Otu00004	*Acinetobacter*	0.00070477
110	Otu00086	*Fusobacterium*	0.00069306
111	Otu00106	*Duganella*	0.00067246
112	Otu00044	*Bradyrhizobium*	0.00067243
113	Otu00065	*Neisseriaceae_unclassified*	0.00067192
114	Otu00124	*Rhodobacteraceae_unclassified*	0.00067156
115	Otu00150	*Ezakiella*	0.00067001
116	Otu00222	*Aquabacterium*	0.00065927
117	Otu00105	*Peptoniphilus*	0.00065815
118	Otu00054	*Stenotrophomonas*	0.00065796
119	Otu00034	*Pasteurellaceae_unclassified*	0.0006539
120	Otu00053	*Aerococcus*	0.00065133
121	Otu00061	*Bacillales_unclassified*	0.00064722
122	Otu00113	*Treponema*	0.00064583
123	Otu00087	*Comamonas*	0.00062164

124	Otu00187	*Actinobacillus*	0.00062127
125	Otu00003	*Pseudomonas*	0.00061036
126	Otu00146	*Lachnospiraceae_unclassified*	0.00060903
127	Otu00131	*Clostridiales_unclassified*	0.0006051
128	Otu00063	*Arthrobacter*	0.00060124
129	Otu00048	*Sphingobium*	0.00059025
130	Otu00033	*Rhodopseudomonas*	0.00055931
131	Otu00039	*Yersinia*	0.00055649
132	Otu00295	*Porphyromonas*	0.00054895
133	Otu00045	*Moraxella*	0.00053853
134	Otu00209	*Ruminococcaceae_unclassified*	0.00051859
135	Otu00159	*Prevotella*	0.00051373
136	Otu00220	*Porphyromonadaceae_unclassified*	0.000513
137	Otu00207	*Micrococcaceae_unclassified*	0.0004654
138	Otu00173	*Pseudomonas*	0.00045048
139	Otu00213	*Peptoniphilus*	0.00044002
140	Otu00023	*Pseudomonas*	0.0004394
141	Otu00134	*Gemella*	0.00042595
142	Otu00415	*Massilia*	0.00036034
143	Otu00305	*Tepidimonas*	0.00033182

Column: 1 – Rank of importance of the OTU in the prediction of a sample belonging to a particular visit; 2- OUT nunber; 3- Bacterial genus; 4- median of the decrease in accuracy (mean decrease in Gini coefficient) when an OTU is removed from the model among the 100 replications of random forest.
