# Supplementary material for: Distinct Microbiotas Are Associated with Different Production Lines in the Cutting Room of a Swine Slaughterhouse
Source: Microorganisms. 2023 Jan 4;11(1):133. doi: 10.3390/microorganisms11010133 (PMC9862343; doi:10.3390/microorganisms11010133)
Supplement: Supplementary file 1 [file microorganisms-11-00133-s001.zip › Table S6 OTUs hierarchized according to their mean importance for the identification of a production line in the random forest production line model.pdf]

Table S6: OTUs hierarchized according to their mean importance for the identification of a production line in the random forest production line model.

Rank	OTUs	Bacterial Genus	Importance
1	Otu00045	*Moraxella*	0,00755239
2	Otu00065	*Neisseriaceae*_unclassified	0,00715058
3	Otu00019	*Macrococcus*	0,0070928
4	Otu00006	*Rothia(92)*	0,00682821
5	Otu00012	*Psychrobacter*	0,00629334
6	Otu00022	*Bacteroides*	0,00586198
7	Otu00033	*Rhodopseudomonas*	0,00524286
8	Otu00034	*Pasteurellaceae*_unclassified	0,00496341
9	Otu00002	*Trueperella*	0,00472953
10	Otu00076	*Anaerococcus*	0,00413672
11	Otu00122	*Moraxellaceae*_unclassified	0,00396747
12	Otu00042	*Chryseobacterium*	0,00376229
13	Otu00020	*Acinetobacter*	0,00349835
14	Otu00059	*Clostridium_sensu_stricto*	0,0033976
15	Otu00249	*Gallicola*	0,00333676
16	Otu00109	*Porphyromonas*	0,00321097
17	Otu00215	*Clostridiales*_unclassified	0,00312908
18	Otu00005	*Peptoniphilus*	0,00301631
19	Otu00014	*Parvimonas*	0,00288531
20	Otu00242	*Corynebacterium*	0,00286469
21	Otu00003	*Pseudomonas*	0,00281765
22	Otu00208	*Ezakiella*	0,00269735
23	Otu00082	*Clostridiaceae_1*_unclassified	0,00267705
24	Otu00011	*Acinetobacter*	0,00267208
25	Otu00120	*Kurthia*	0,00265106
26	Otu00175	*Dechloromonas*	0,00259474
27	Otu00008	*Staphylococcus*	0,00259435
28	Otu00047	*Psychrobacter*	0,00256874
29	Otu00055	*Carnobacterium*	0,00255095
30	Otu00018	*Peptostreptococcus*	0,0025103
31	Otu00232	*Streptococcus*	0,00250622
32	Otu00281	*Desulfovibrio*	0,00239995
33	Otu00063	*Arthrobacter*	0,00239343
34	Otu00105	*Peptoniphilus*	0,00236185
35	Otu00079	*Porphyromonas*	0,00233519
36	Otu00141	*Peptoniphilus*	0,00232642
37	Otu00258	*Bacteroides*	0,00225725
38	Otu00112	*Mycoplasma*	0,00218713
39	Otu00453	*Bacteroidetes*_unclassified	0,00217711

40	Otu00196	*Soonwooa*	0,00214222
41	Otu00054	*Stenotrophomonas*	0,00213316
42	Otu00010	*Aerococcaceae*_unclassified	0,00212038
43	Otu00201	*Flavobacteriaceae*_unclassified	0,00205318
44	Otu00023	*Pseudomonas*	0,00203361
45	Otu00188	*Bacteroidales*_unclassified	0,00203197
46	Otu00027	*Janthinobacterium*	0,00199127
47	Otu00458	*Mannheimia*	0,00195721
48	Otu00009	*Bacteroides*	0,00192371
49	Otu00071	*Enterococcus*	0,00192169
50	Otu00515	*Lactobacillales*_unclassified	0,00187147
51	Otu00090	*Atopobium*	0,00182341
52	Otu00016	*Clostridium_sensu_stricto*	0,00173585
53	Otu00025	*Chryseobacterium*	0,00168543
54	Otu00062	*Aeromonas*	0,00168175
55	Otu00080	*Helcococcus*	0,0016784
56	Otu00166	*Paludibacter*	0,00166812
57	Otu00409	*Leucobacter*	0,00166659
58	Otu00056	*Phenylobacterium*	0,00165141
59	Otu00393	*Acidaminococcus*	0,00161787
60	Otu00163	*Finegoldia*	0,00160598
61	Otu00127	*Exiguobacterium*	0,00160188
62	Otu00152	*Porphyromonas*	0,00158718
63	Otu00161	*Bacteroidales*_unclassified	0,00158152
64	Otu00490	*Coriobacteriaceae*_unclassified	0,00153199
65	Otu00615	*Desulfovibrio*	0,00152779
66	Otu00461	*Deinococcus*	0,00145036
67	Otu00240	*Prevotella*	0,00143422
68	Otu00081	*Corynebacterium*	0,00142584
69	Otu00285	*Rhodocyclaceae*_unclassified	0,00140776
70	Otu00140	*Peptoniphilaceae*_unclassified	0,00140308
71	Otu00373	*Propionivibrio*	0,00140222
72	Otu00477	*Sulfurospirillum*	0,00138963
73	Otu00017	*Bacteroides*	0,00138454
74	Otu00262	*Lactococcus*	0,0013752
75	Otu00001	*Fusobacterium*	0,00135314
76	Otu00157	*Clostridiales_Incertae_Sedis_XI*_unclassified	0,00134758
77	Otu00041	*Terrisporobacter*	0,00131979
78	Otu00375	*Bacteroidales*_unclassified	0,00131511
79	Otu00199	*Bacteroides*	0,00129308
80	Otu00044	*Bradyrhizobium*	0,00128157
81	Otu00039	*Yersinia*	0,00126017
82	Otu00028	*Porphyromonas*	0,00125809

83	Otu00251	*Corynebacterium*	0,001254
84	Otu00052	*Porphyromonas*	0,00123527
85	Otu00070	*Sphingomonas*	0,0012286
86	Otu00099	*Lactobacillus*	0,00122703
87	Otu00013	*Porphyromonas*	0,0012258
88	Otu00316	*Flavobacteriaceae*_unclassified	0,00121006
89	Otu00032	*Porphyromonas*	0,00120761
90	Otu00206	*Microvirgula*	0,00118001
91	Otu00155	*Flavobacteriaceae*_unclassified	0,00116817
92	Otu00069	*Lachnospiraceae*_unclassified	0,00116382
93	Otu00058	*Turicibacter*	0,00116148
94	Otu00264	*Bacteroides*	0,0011565
95	Otu00150	*Ezakiella*	0,00114923
96	Otu00078	*Polaromonas*	0,00111556
97	Otu00391	*Vitreoscilla*	0,00110177
98	Otu00102	*Fusobacterium*	0,00108047
99	Otu00097	*Eubacterium*	0,00107498
100	Otu00576	*Petrimonas*	0,00107408
101	Otu00406	*Actinomycetaceae*_unclassified	0,00106972
102	Otu00172	*Cloacibacterium*	0,00104756
103	Otu00259	*Moraxella*	0,00103668
104	Otu00447	*Bacteria_unclassified*	0,00103381
105	Otu00154	*Ruminococcaceae*_unclassified	0,00103342
106	Otu00664	*Synergistaceae*_unclassified	0,00103071
107	Otu00317	*Catenibacterium*	0,0010273
108	Otu00136	*Clostridium_sensu_stricto*	0,00102662
109	Otu00237	*Streptococcus*	0,00101221
110	Otu00606	*Synergistaceae*_unclassified	0,00098553
111	Otu00298	*Clostridiales*_unclassified	0,00097175
112	Otu00049	*Prevotella*	0,00095805
113	Otu00197	*Campylobacter*	0,00095794
114	Otu00537	*Brachymonas*	0,00095132
115	Otu00015	*Aerococcaceae*_unclassified	0,00095121
116	Otu00110	*Coriobacteriaceae*_unclassified	0,00095112
117	Otu00454	*Porphyromonas*	0,00094579
118	Otu00106	*Duganella*	0,00094553
119	Otu00203	*Clostridium_sensu_stricto*	0,00093991
120	Otu00359	*Bacteroidetes*_unclassified	0,00092562
121	Otu00035	*Fusobacterium*	0,00090469
122	Otu00104	*Helcococcus*	0,00090286
123	Otu00186	*Peptostreptococcus*	0,00089187
124	Otu00193	*Porphyromonas*	0,00088142
125	Otu00211	*Phascolarctobacterium*	0,00087206

126	Otu00130	*Facklamia*	0,00086857
127	Otu00526	*Lachnospiraceae*_unclassified	0,00086839
128	Otu00089	*Porphyromonadaceae*_unclassified	0,0008669
129	Otu00075	*Romboutsia*	0,00084347
130	Otu00480	*Flavobacterium*	0,00084301
131	Otu00225	*Fastidiosipila*	0,00083872
132	Otu00510	*Telmatospirillum*	0,00081654
133	Otu00253	*Lachnospiraceae*_unclassified	0,00081054
134	Otu00138	*Flavobacterium*	0,00081005
135	Otu00142	*Brevibacterium*	0,00080723
136	Otu00207	*Micrococcaceae*_unclassified	0,00080632
137	Otu00624	*Synergistaceae*_unclassified	0,00079537
138	Otu00107	*Clostridiales*_unclassified	0,00078719
139	Otu00093	*Psychrobacter*	0,00078295
140	Otu00159	*Prevotella*	0,0007544
141	Otu00085	*Bacteria*_unclassified	0,00075285
142	Otu00337	*Morganella*	0,00074823
143	Otu00118	*Bacteria*_unclassified	0,00073945
144	Otu00518	*Alphaproteobacteria*_unclassified	0,00073545
145	Otu00260	*Bacteria*_unclassified	0,00072761
146	Otu00216	*Anaerococcus*	0,00070862
147	Otu00116	*Mycobacterium*	0,00070682
148	Otu00202	*Anaerococcus*	0,000704
149	Otu00482	*Bacteroidales*_unclassified	0,00070046
150	Otu00115	*Mycoplasma*	0,00069574
151	Otu00179	*Pseudomonas*	0,00068442
152	Otu00322	*Azonexus*	0,00068263
153	Otu00486	*Flavobacterium*	0,00068001
154	Otu00231	*Sphingopyxis*	0,00067664
155	Otu00088	*Streptococcus*	0,00067636
156	Otu00029	*Streptococcus*	0,00067517
157	Otu00250	*Clostridiales_Incertae_Sedis_XI*_unclassified	0,00067288
158	Otu00468	*Pseudomonas*	0,00066168
159	Otu00351	*Ruminococcaceae*_unclassified	0,00064984
160	Otu00516	*Macellibacteroides*	0,00064831
161	Otu00046	*Escherichia/Shigella*	0,00063966
162	Otu00217	*Propionispira*	0,00063472
163	Otu00347	*Prevotella*	0,00062642
164	Otu00031	*Epilithonimonas*	0,00060814
165	Otu00131	*Clostridiales*_unclassified	0,00060486
166	Otu00703	*Lachnospiraceae*_unclassified	0,00059495
167	Otu00400	*Clostridiales*_unclassified	0,00059451
168	Otu00068	*Actinomyces*	0,00057723

169	Otu00363	*Dysgonomonas*	0,000569
170	Otu00503	*Proteiniclasticum*	0,00056821
171	Otu00134	*Gemella*	0,00056605
172	Otu00030	*Bacteroides*	0,0005535
173	Otu00313	*Lactobacillus*	0,0005428
174	Otu00026	*Salmonella*	0,00053659
175	Otu00132	*Lactococcus*	0,00051584
176	Otu00611	*Bacteroidales*_unclassified	0,00050892
177	Otu00233	*Campylobacter*	0,0005029
178	Otu00164	*Actinomycetaceae*_unclassified	0,00049325
179	Otu00153	*Ruminococcaceae*_unclassified	0,00048525
180	Otu00603	*Cellulomonas*	0,00048046
181	Otu00223	*Porphyromonas*	0,00043022
182	Otu00581	*Veillonellaceae*_unclassified	0,00040995

Column: 1 – Rank of importance of the OTU in the prediction of a sample belonging to a particular production line; 2- OTU number; 3- Bacterial genus; 4- median of the decrease in accuracy (mean decrease in Gini coefficient) when an OTU is removed from the model among the 100 replications of random forest.
